# Frontier Advances in Wind-Driven Triboelectric Nanogenerators for Realistic Wind Environments: Scenario-Oriented Architecture Design, System Integration, and Critical Assessment

**DOI:** 10.3390/mi17091024

**Published:** 2026-08-28

**Authors:** Mingkang Zhu, Jing Wu, Guangxi Li, Zikang Li, Hao Liu, Kaicheng Yu, Sheng Zhang, Chao Wang

**Affiliations:** 1 School of Mechanical Engineering, Dongguan University of Technology, Dongguan 523808, China; 2 Shien-Ming Wu School of Intelligent Engineering, South China University of Technology, Guangzhou 511442, China; 3 Dongguan Key Laboratory of Intelligent Bionic Robot Technology and System, Dongguan 523808, China; 4 School of Materials Science and Engineering, Anhui University of Science and Technology, Huainan 232001, China

**Keywords:** triboelectric nanogenerator, wind energy harvesting, realistic wind environments, power management and system integration, reliability and standardization

## Abstract

Triboelectric nanogenerators (TENGs) offer promising opportunities for distributed wind energy harvesting owing to their low-speed responsiveness, structural flexibility, and adaptability to non-stationary airflow. This review examines wind-driven TENGs from the perspective of realistic wind-field constraints, focusing on three representative scenarios: urban micro-winds, offshore wind–wave environments, and low-altitude complex flows. Scenario-specific advances in device architectures, materials and interfaces, environmental protection, power management, and system integration are systematically reviewed. Representative devices are further quantitatively compared in terms of wind-speed range, activation threshold, electrical output, power density, durability, and system-level energy delivery. Particular attention is given to inconsistent definitions of cut-in wind speed, output normalization, electrical loading, and validation conditions that limit cross-study comparison. Field-validation evidence is assessed from controlled laboratory tests to long-term field operation. Key challenges involving usable regulated energy, environmental reliability, lifetime prediction, array scaling, sustainability, and deployment economics are critically discussed. Finally, five grand challenges with actionable milestones are proposed to facilitate the transition of wind-driven TENGs from laboratory prototypes toward deployable distributed micro-energy systems.

## 1. Introduction

### 1.1. Evolution and Limitations of Conventional Wind Energy Technologies

Wind energy is among the fastest-growing renewable energy sources worldwide. For decades, large-scale electricity generation has been dominated by horizontal-axis wind turbines [1] and vertical-axis wind turbines [2]. These technologies are primarily designed to maximize energy capture under relatively high wind speeds and steady inflow conditions, and to convert mechanical power into grid-compatible electricity. In recent years, however, the rapid development of floating offshore wind farms, low-altitude wind exploitation, and distributed sensing networks has exposed the practical boundaries of conventional turbines in built environments, marginal offshore platforms, and low-altitude complex flows.

Specifically, traditional wind turbines often suffer from a high cut-in wind speed, pronounced output fluctuations, accelerated structural fatigue, and strict space requirements when operated under low wind speeds and highly turbulent conditions [3]. In urban areas, building-induced blockage generates strong wakes and intermittent turbulence, resulting in highly unsteady wind fields. Offshore environments introduce additional challenges such as salt spray corrosion, biofouling, and long-term unattended operation. In the atmospheric boundary layer at low altitude, rapid wind shear and wind-direction fluctuations further increase the difficulty of stable operation for rotating generators. Therefore, wind energy harvesting is increasingly shifting from “centralized power generation in strong winds” toward “in situ power supply in complex micro-wind fields”, to better match the energy demands of distributed sensors and edge devices [4].

### 1.2. Value of Triboelectric Nanogenerators for Wind Energy Harvesting

In 2012, Zhong Lin Wang’s group first proposed the concept of triboelectric nanogenerators (TENGs) [5]. TENGs rely on the coupled effects of triboelectrification and electrostatic induction. When two materials with different electron affinities periodically contact–separate or slide against each other, mechanical energy can be directly converted into electricity [6]. Since their introduction, TENGs have demonstrated distinctive advantages for mechanical energy harvesting. Compared with electromagnetic and piezoelectric generators, TENGs are typically lightweight, structurally versatile, capable of high output voltage, and effective under low-frequency excitations [7]. Importantly, their operating mechanism aligns well with small-amplitude vibrations, low-speed airflow, and non-stationary mechanical stimuli commonly encountered in realistic wind fields [8].

From an application perspective, wind-driven TENGs offer three key benefits. First, their low activation threshold enables harvesting of low-speed or intermittent airflow that is difficult for conventional turbines to utilize effectively [9,10]. Second, their flexibility and integrability facilitate tight coupling with building envelopes, ventilation ducts, offshore buoys, and low-altitude platforms [11,12]. Third, their pulsed high-voltage output can be conditioned through energy storage and power-management circuits to support low-power loads such as sensing, communication, and monitoring modules [13,14]. Accordingly, wind-driven TENGs should be viewed as a complementary solution for distributed micro-energy systems, providing localized power for low-power electronics, self-powered sensor nodes, and edge computing terminals rather than directly competing with utility-scale wind turbines.

More fundamentally, TENG-enabled wind harvesting represents a paradigm shift beyond incremental technological improvement. (i) Scenario redefinition: from high-altitude, relatively stable wind resources targeted by conventional turbines to distributed wind fields that are typically underexploited, such as building facades and rooftops, deep-sea buoys, and tethered low-altitude platforms. Although these resources are smaller in magnitude, they are spatially co-located with end devices (e.g., IoT sensors and wireless nodes). (ii) Metric redefinition: from maximizing power density and the power coefficient to prioritizing low cut-in wind speed, tolerance to turbulence-induced fluctuations, long-life, and low-maintenance operation. (iii) Energy-use redefinition: from centralized generation and grid integration to in situ harvesting, storage, and utilization—directly powering local low-power devices without reliance on long-distance transmission infrastructure, consistent with the energy requirements of distributed sensing and edge computing.

### 1.3. New Perspective and Organization of This Review

Most existing reviews on wind-driven TENGs are organized around device structures or operation modes [15,16], such as contact–separation, single-electrode, lateral-sliding, and freestanding triboelectric-layer configurations [17,18]. While this classification is useful for clarifying working principles, it often provides limited discussion of real-world wind characteristics, environmental constraints, and system-level integration.

In practice, wind environments differ substantially across application scenarios. Urban winds are typically characterized by low mean speeds and strong turbulence; offshore conditions involve salt spray, corrosion, and wind–wave coupling; and low-altitude environments cover a broad wind-speed range with complex wind shear. These differences necessitate scenario-specific choices in structural configuration, material and interfacial engineering, packaging strategies, and power management.

**Figure 1:**
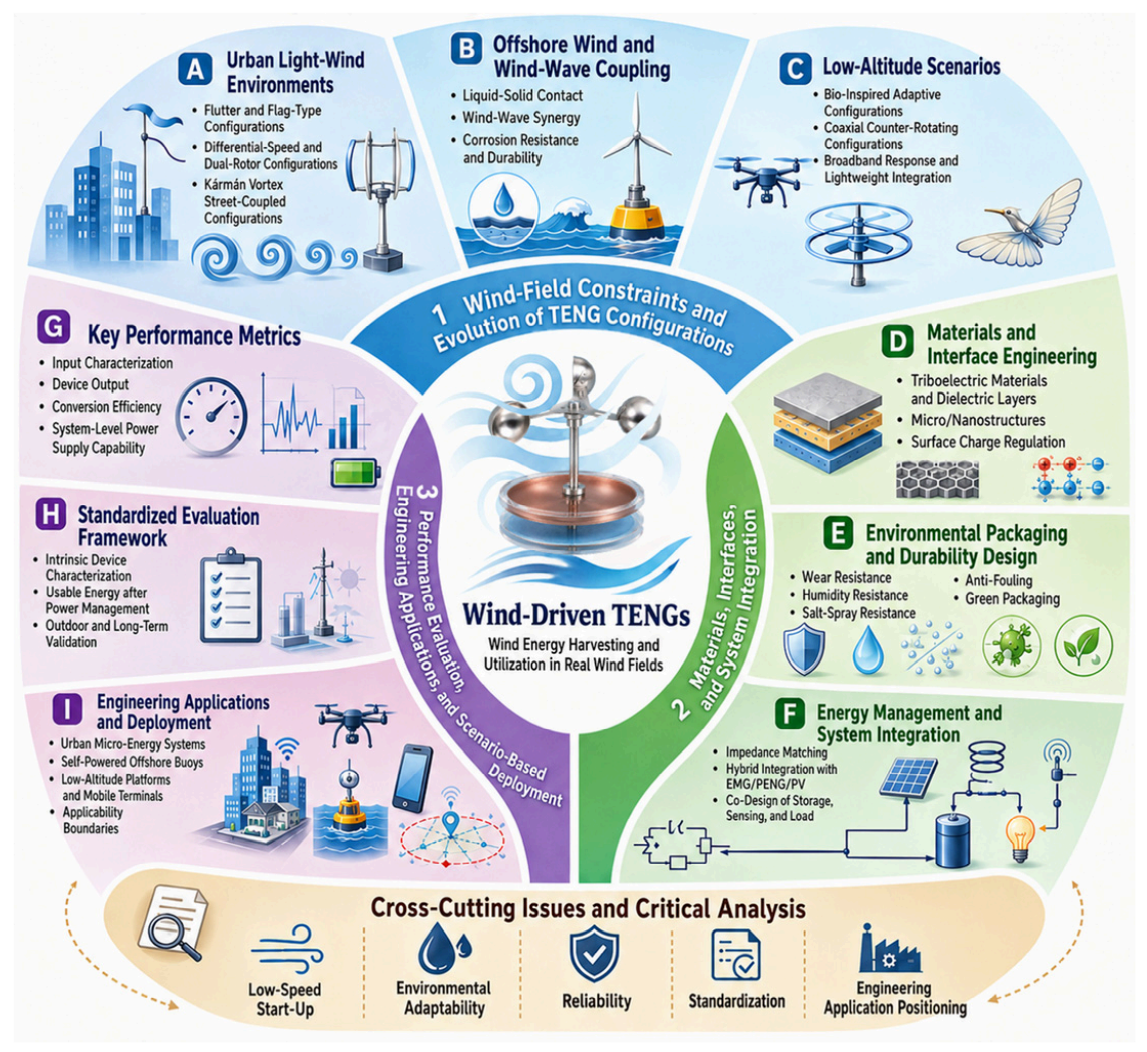
Scenario-oriented framework of wind-driven TENGs for realistic wind environments, covering urban micro-winds, offshore wind–wave coupled environments, and low-altitude complex wind fields, together with architecture design, materials/interfaces, system integration, performance evaluation, and engineering deployment.

Therefore, this review is structured around constraints imposed by realistic wind fields rather than a simple enumeration of operation modes. We discuss wind-driven TENGs across three representative application domains: urban micro-wind environments, offshore wind–wave coupled settings, and low-altitude complex wind fields. Within this framework, we further provide a critical analysis focusing on performance evaluation, standardized testing, array-based scaling, lifetime prediction, and sustainability. Our goal is to establish an application-oriented analytical framework that clarifies the current applicability limits of wind-driven TENGs and outlines key directions for future development. The overall structure of this review is illustrated in Figure 1.

## 2. Wind-Field Constraints and the Evolution of TENG Architecture Design

### 2.1. Urban Micro-Winds: Low-Speed Harvesting Under Turbulent Conditions

Wind resources in urban environments are inherently non-stationary. The wind-speed distribution is jointly shaped by building blockage, street-canyon effects, ventilation corridors, and local thermal convection. As a result, urban wind fields are typically characterized by low mean wind speeds, high turbulence intensity, and strong stochastic fluctuations. Compared with open plains or high-altitude winds, urban flows resemble a multiscale, coupled system. On the one hand, wind speeds near the building boundary layer are usually low, which favors distributed deployment of micro-energy harvesters. On the other hand, wakes, vortex shedding, and transient pulsations make the wind energy input markedly non-periodic, imposing stricter requirements on both cut-in capability and output stability.

For conventional rotary wind turbines, urban wind is often far from an ideal operating condition. Because these turbines rely on relatively steady inflow and sufficient rotor-swept area, low-speed and highly turbulent winds substantially reduce aerodynamic efficiency and exacerbate mechanical fatigue and noise. In contrast, TENGs are better suited to urban micro-wind scenarios due to their low cut-in threshold, strong nonlinear response to unsteady excitation, and potential for lightweight and flexible deployment. In spatially constrained locations with locally intensified flow—such as ventilation ducts, facade edges, rooftop separation zones, and the underside of bridges—TENGs can harvest energy in situ by leveraging pulsating airflow or vortex-induced excitation. This capability enables localized power supply for environmental monitoring, structural health monitoring (SHM), and low-power sensor nodes.

Existing studies have developed multiple structural routes to accommodate diverse wind conditions, including flutter-based, flag-type, rotary, vortex-induced, liquid–solid contact, wind–wave synergistic, and bioinspired adaptive configurations. Consequently, in urban micro-wind environments, design priorities have shifted from pursuing higher peak output to emphasizing low-speed responsiveness, tolerance to turbulence-induced disturbances, structural compactness, and long-term operational robustness. In essence, urban wind utilization is better framed as a “micro-energy adaptation problem in complex flows” rather than a conventional large-scale power-generation problem.

#### 2.1.1. Flutter-Based and Flag-Type Configurations

**Figure 2:**
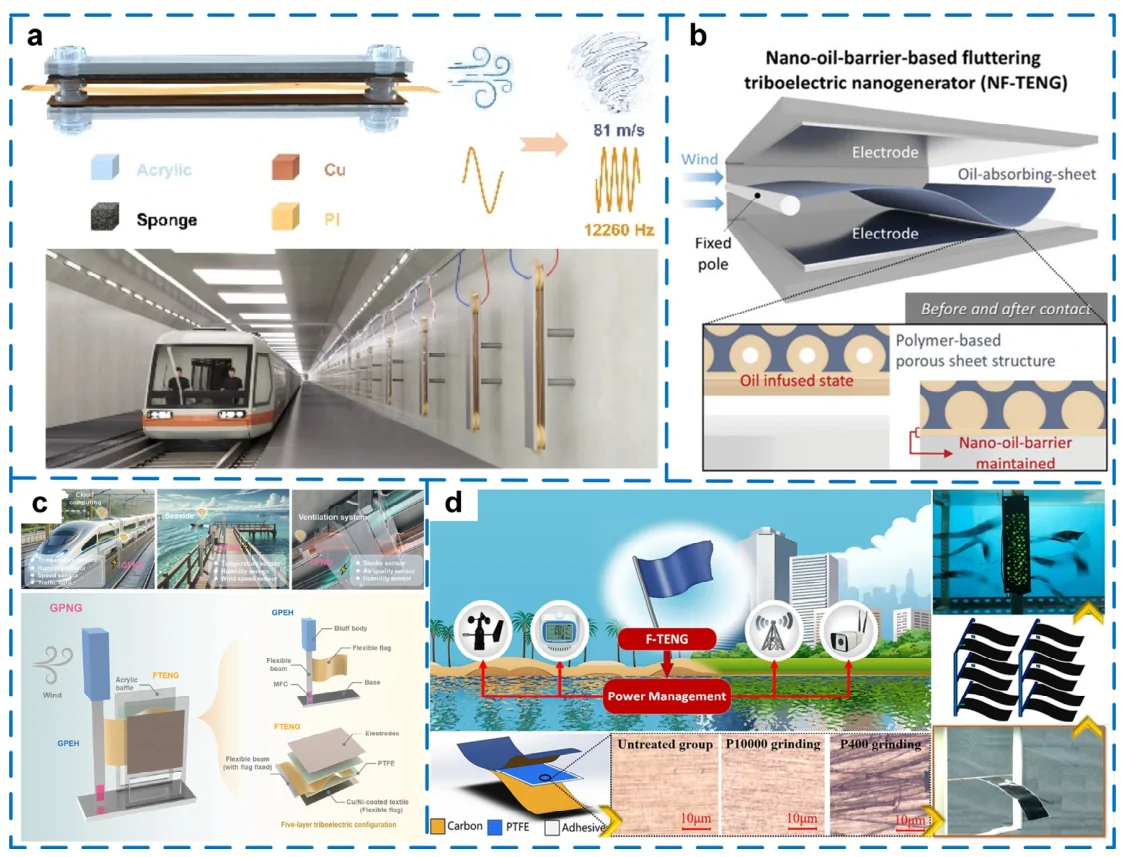
Flutter-based and flag-type TENGs for wind energy harvesting: (**a**) Double-ended fixed fluttering-structure TENG. Reproduced with permission from ref. [20]. Copyright (2025) Royal Society of Chemistry. (**b**) Nano-oil-barrier-based fluttering TENG. Reproduced with permission from ref. [21], used with permission of the Creative Commons CC-BY. (**c**) Galloping–flutter coupled TENG. Reproduced with permission from ref. [22]. Copyright (2024) John Wiley and Sons. (**d**) Array of flag-type TENGs [25].

In urban micro-wind scenarios, where wind speeds are low, directions are random, and turbulence is strong, flutter-based [19,20,21,22] and flag-type TENGs [23,24,25] are among the most widely adopted designs. Representative examples include a double-ended fixed fluttering TENG (Figure 2a), a nano-oil-barrier-based fluttering TENG for improved interfacial durability (Figure 2b), a galloping–flutter coupled configuration (Figure 2c), and a flag-type TENG array for distributed wind harvesting (Figure 2d). The core idea is to use self-excited oscillations of a flexible film under airflow. Fluid kinetic energy is first converted into periodic mechanical motion, and then into electrical output through triboelectrification and electrostatic induction. These configurations feature low cut-in wind speed, simple structures, and straightforward integration with building surfaces or air outlets, making them particularly suitable for harvesting energy in local separated-flow regions.

Compared with rigid rotary structures, flutter-based TENGs better tolerate wind-direction variations and can operate continuously under wake-driven or duct-induced pulsating flows. Nevertheless, they often face limitations such as constrained vibration amplitude, long-term wear, and insufficient output consistency. Recent efforts have therefore focused on improving material durability, optimizing flexible support structures, and introducing non-contact or weak-contact modes to enhance outdoor serviceability.

Flag-type TENGs further exploit the flapping dynamics of a flexible membrane in airflow to generate periodic triboelectric output. Under weak winds, the membrane mass, length, tension state, and boundary constraints strongly affect the vibration frequency and effective contact amplitude. Overly soft designs may lead to ineffective flapping without stable contact, whereas overly stiff designs raise the cut-in wind speed. Therefore, rational tuning of the elastic modulus, membrane geometry, and support gap is critical for achieving low-speed activation and stable output.

#### 2.1.2. Differential-Rotation and Dual-Rotor Configurations

**Figure 3:**
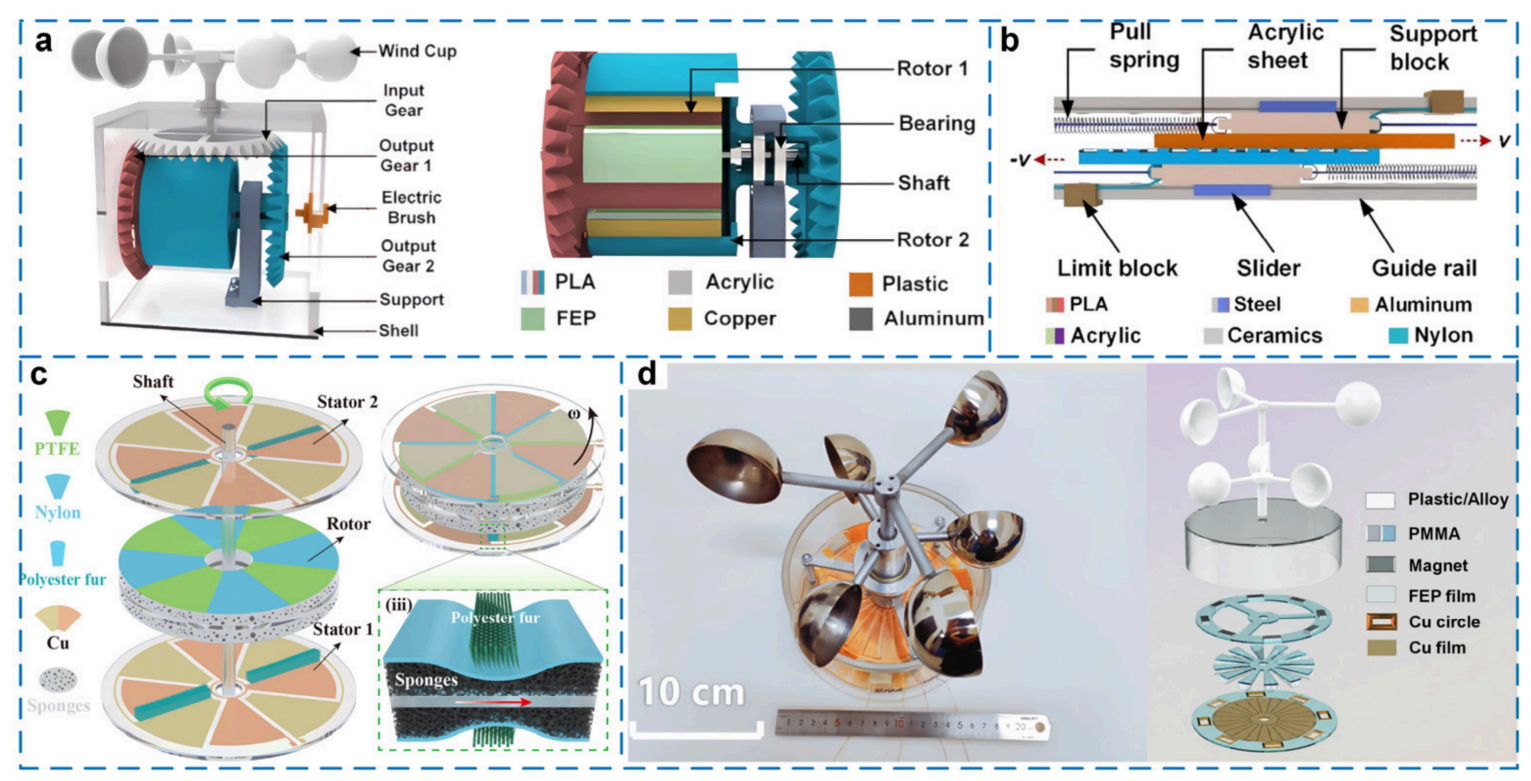
Differential-rotation and dual-rotor TENG architectures for wind energy harvesting: (**a**) Rotation differential TENG. Reproduced with permission from ref. [27]. Copyright (2024) IOP Publishing. (**b**) Differential TENG. Reproduced with permission from ref. [28]. Copyright (2022) IOP Publishing. (**c**) Ternary four-phase soft–soft contact TENG. Reproduced with permission from ref. [29], used with permission of the Creative Commons CC-BY. (**d**) TENG driven by dual coaxial rotating-shaft wind cups. Reproduced with permission from ref. [30]. Copyright (2022) John Wiley and Sons.

In urban and mountainous settings, frequent wind-direction changes make broadband-adaptive or multi-axis rotation advantageous [26]. Conventional wind harvesters often require yaw mechanisms or active direction tracking, whereas differential-rotation and dual-rotor TENGs can reduce directional dependence by exploiting relative motion between rotors. By combining multiple rotating units, differential transmission, or counter-rotating triboelectric layers, these architectures convert wind-driven rotation into higher-frequency contact or sliding processes, thereby improving output stability [27,28,29,30,31,32,33]. Representative designs include a rotation-differential TENG (Figure 3a), a differential TENG using relative mechanical motion (Figure 3b), a ternary four-phase soft–soft contact configuration (Figure 3c), and a TENG driven by dual coaxial wind-cup shafts (Figure 3d).

Such designs provide two main benefits. First, relative motion between rotors can improve airflow utilization. Second, mechanical coupling can mitigate performance fluctuations caused by abrupt wind-direction changes. In urban environments, these configurations are particularly suitable for locations with unstable wind direction but quasi-periodic local speed variations, such as passages between buildings, edges of bridge structures, and rooftops of high-rise buildings. Another practical advantage of differential-rotation designs is “mechanical rectification”: converting inherently non-periodic airflow disturbances into more regular contact–separation motion, which improves output predictability. For low-power sensor nodes that require continuous energy supply, this stability can be more important than instantaneous peak power.

However, differential and dual-rotor designs also introduce new engineering challenges. Multi-rotor systems generally demand more complex supports and bearing assemblies, potentially increasing friction losses, noise, and maintenance requirements. High-speed sliding contact may also accelerate tribolayer wear and charge decay. Therefore, these architectures require careful trade-offs among enhanced relative motion, reduced mechanical wear, and improved system reliability.

#### 2.1.3. Karman Vortex Street-Coupled Configurations

**Figure 4:**
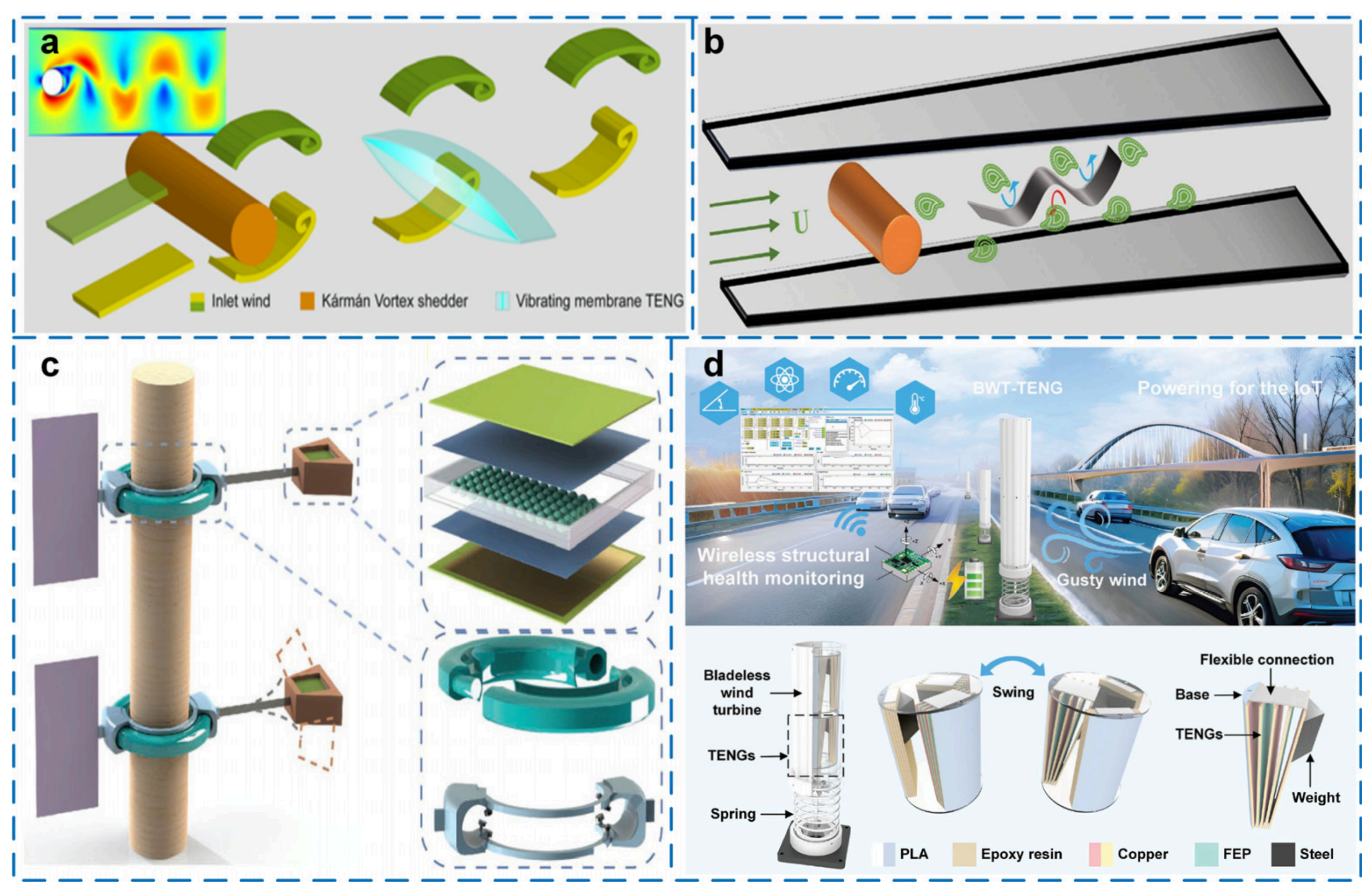
Wind-driven Karman vortex street–excited TENGs for wind energy harvesting: (**a**) Karman vortex street-driven membrane TENG [33]. (**b**) Wake-flow-induced flutter TENG. Reproduced with permission from ref. [34]. Copyright (2026) John Wiley and Sons. (**c**) Omnidirectional fluid-induced vibration TENG based on a cantilever structure. Reproduced with permission from ref. [35]. Copyright (2023) John Wiley and Sons. (**d**) Bladeless wind-turbine TENG. Reproduced with permission from ref. [36]. Copyright (2024) John Wiley and Sons.

Karman vortex street-coupled designs utilize periodic vortex shedding in bluff-body flows to transform otherwise disordered turbulent disturbances into regular vibrational excitation, thereby driving TENG output [33,34,35,36,37,38]. Representative vortex-coupled architectures include a Karman-vortex-driven membrane TENG (Figure 4a), a wake-flow-induced flutter TENG (Figure 4b), an omnidirectional fluid-induced vibration TENG (Figure 4c), and a bladeless wind-turbine TENG for gust-energy harvesting (Figure 4d). Compared with structures that rely directly on wind pressure, vortex-induced vibration can produce relatively stable periodic responses within a certain wind-speed range. This makes it attractive for wind-speed monitoring, external-flow sensing around pipelines, and micro-wind harvesting near bridges. By optimizing the bluff-body geometry, membrane thickness, and boundary conditions, TENGs can achieve self-excited operation at lower wind speeds, substantially expanding the exploitable range of low-speed wind resources.

Overall, the architectural evolution in urban micro-wind scenarios shows a clear shift—from “passively responding to airflow” toward “actively leveraging flow instabilities.” In this context, the design philosophy is no longer to suppress turbulence as much as possible, but to treat it as an accessible and exploitable energy source.

### 2.2. Offshore Wind and Wind–Wave Coupled Environments: Architectures for Synergistic Energy Harvesting

Offshore wind fields offer advantages such as higher wind speeds, open space, and relatively persistent resources. Meanwhile, they impose stringent engineering constraints, including salt spray, high humidity, severe corrosion, marine biofouling, and long-term unattended operation. For ocean platforms, buoy systems, nearshore monitoring devices, and marine infrastructures, the energy supply must not only support low-power sensing and communication, but also deliver long-term reliability, resistance to environmental degradation, and low maintenance cost.

In this context, wind-driven TENGs provide value in two major aspects. First, TENGs can operate synergistically with low-speed airflow, wave-induced disturbances, and platform motion in marine environments, enabling multi-source coupled harvesting of wind energy, wave energy, and even mechanical rocking energy [39]. Second, the structural versatility of TENGs allows stability enhancement via robust encapsulation, material substitution, liquid–solid interface engineering, and anti-corrosion coatings, thereby extending service life and improving practical usability [40].

Compared with terrestrial deployment, offshore applications place greater emphasis on system-level performance rather than single-device metrics. In other words, an offshore TENG must not only exhibit efficient electromechanical conversion, but also achieve comprehensive robustness in packaging integrity, salt-fog tolerance, anti-biofouling capability, and energy-storage matching. In self-powered offshore buoys, marine environmental monitoring platforms, and SHM of ocean engineering systems, TENGs are typically integrated with supercapacitors, low-power communication modules, and environmental sensors to form complete self-sustained power units. Accordingly, evaluation criteria should expand from “single-cycle output” to “long-term operational stability” and “system-level energy balance”.

Therefore, offshore wind-driven TENGs are better positioned as distributed micro-power solutions designed to withstand environmental disturbances. Their primary mission is not to replace conventional offshore wind turbines, but to provide sustained energy for small-power, low-maintenance, autonomous devices in marine settings.

#### 2.2.1. Liquid–Solid Contact Configurations

**Figure 5:**
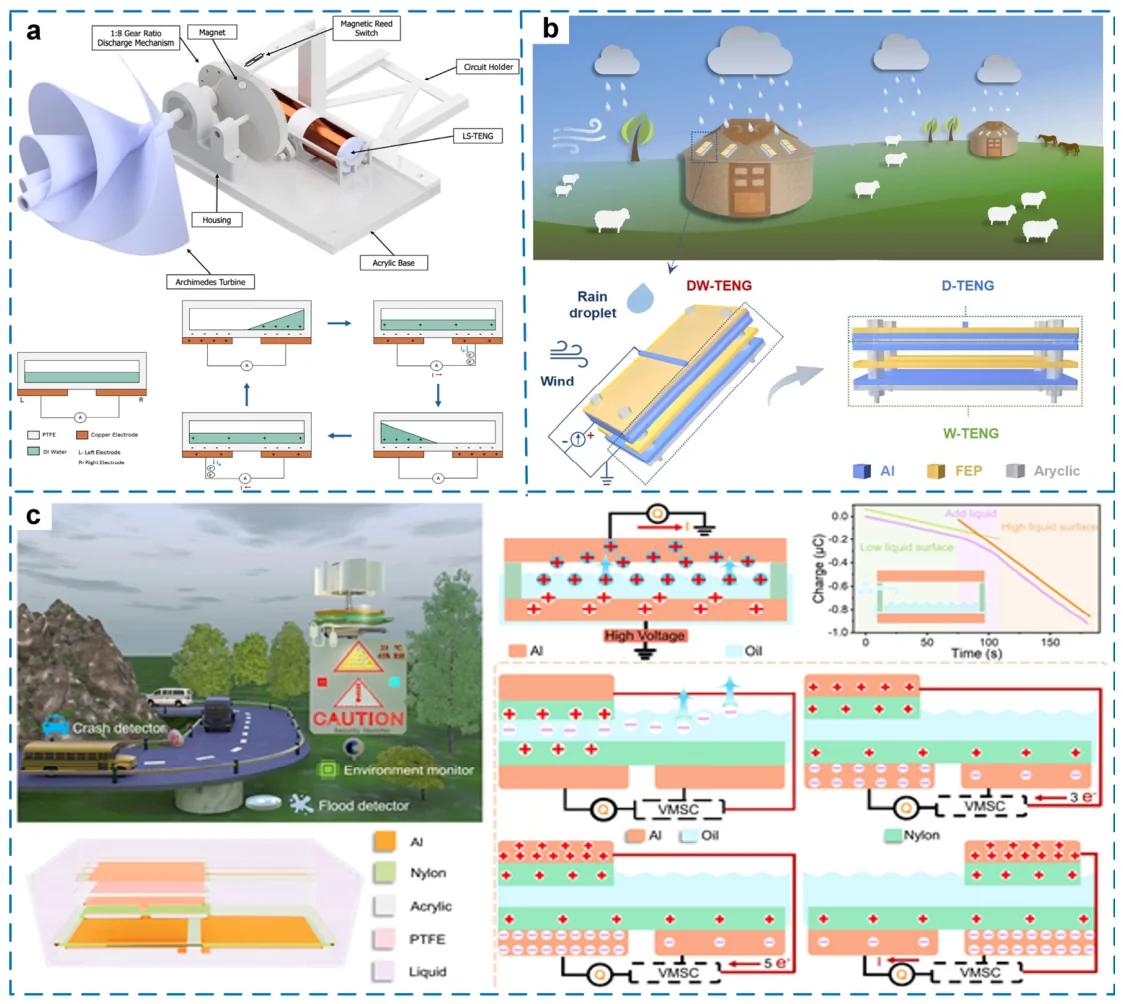
Liquid–solid contact TENGs coupled with wind energy harvesting: (**a**) Wind turbine-driven liquid–solid TENG. Reproduced with permission from ref. [41]. Copyright (2025) John Wiley and Sons. (**b**) Hybridized TENG integrating a droplet-driven TENG and a wind-driven TENG [42]. (**c**) Self-excited liquid suspension TENG. Reproduced with permission from ref. [43]. Copyright (2022) John Wiley and Sons.

The emergence of liquid–solid contact TENGs coupled with wind energy harvesting [41,42,43,44] indicates a shift in offshore-oriented designs from conventional solid–solid interfaces toward more environment-adaptive approaches. As representative examples, Figure 5a shows a wind-turbine-driven liquid–solid TENG, Figure 5b integrates droplet- and wind-driven TENG units for multi-source harvesting, and Figure 5c employs a self-excited liquid-suspension configuration. Compared with traditional contact-based structures, liquid–solid designs can exploit the dynamic spreading and recovery of liquid interfaces to reduce mechanical wear and, to some extent, alleviate interfacial degradation under salt-fog exposure. For seawater-related applications, appropriate material selection and encapsulation can even enable controlled utilization of conductive liquids or seawater interfaces.

While liquid–solid configurations offer improved wear tolerance and environmental adaptability, further optimization is needed in output stability, liquid management, and long-term sealing integrity. Particularly for offshore platforms and buoy systems, preventing performance decay caused by liquid evaporation, leakage, or contamination remains a key barrier to engineering-scale deployment.

#### 2.2.2. Wind–Wave Synergistic Configurations

**Figure 6:**
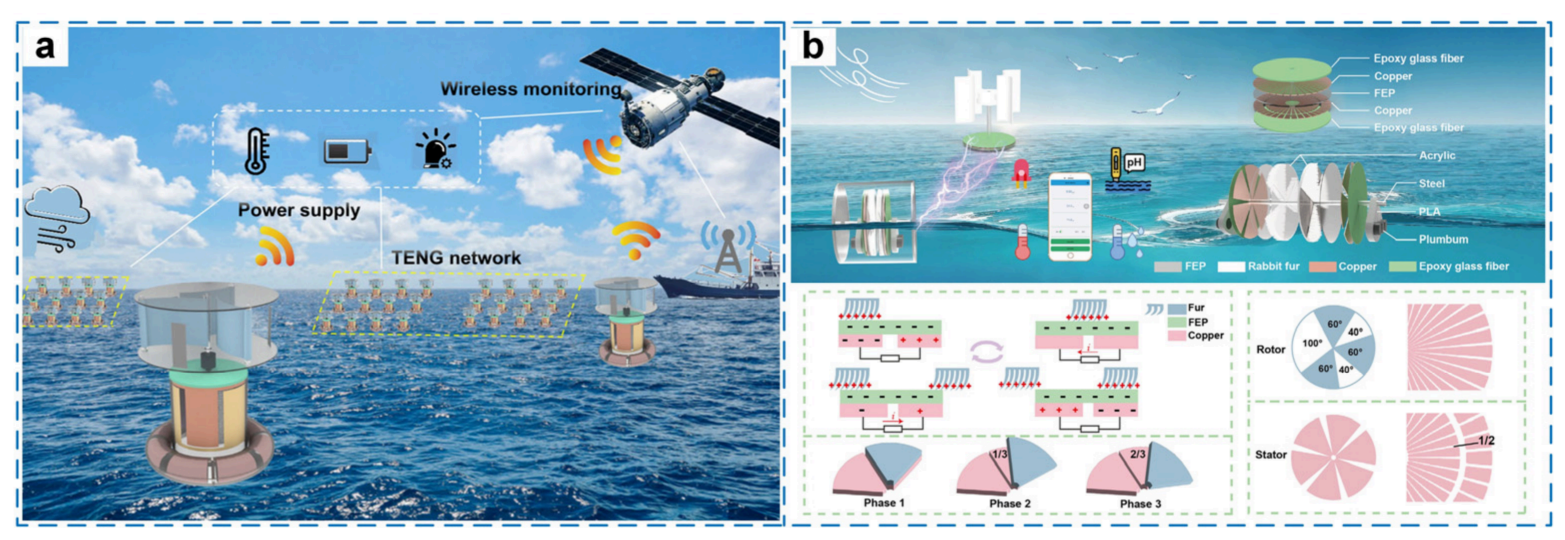
Wind–wave synergistic TENG architectures: (**a**) Wind and wave energy harvester based on a TENG. Reproduced with permission from ref. [45]. Copyright (2024) John Wiley and Sons. (**b**) Hybrid blue-energy harvesting device based on a constant-voltage TENG. Reproduced with permission from ref. [46]. Copyright (2026) John Wiley and Sons.

Wind–wave synergistic designs directly reflect the “coexistence of multiple energy sources” in marine environments. Wind and wave energy often exhibit spatiotemporal correlation; relying on a single source can lead to intermittent supply, whereas synergistic harvesting can improve output continuity and stability. Because wind and waves frequently coexist on offshore or lacustrine monitoring platforms, wind–wave synergistic TENGs have high practical relevance [45,46,47,48,49,50]. Representative wind–wave synergistic systems include an integrated TENG for combined wind and wave energy harvesting (Figure 6a) and a hybrid blue-energy harvesting device based on a constant-voltage TENG (Figure 6b). Such systems typically employ coaxial dual rotors, buoy–structure coupling, or multi-degree-of-freedom mechanical units to capture wind and wave energy simultaneously.

From a system-design viewpoint, the main significance of wind–wave synergy is not merely increasing total power, but enhancing supply reliability through energy complementarity. For long-term offshore deployments such as monitoring buoys, meteorological stations, and inspection platforms, continuity and stability of the energy supply are often more critical than peak output. Wind–wave synergy is therefore a direction that warrants sustained research efforts in offshore TENG development.

Nevertheless, synergistic structures face the challenge of complex input coupling. Wind and waves differ in frequency, direction, and amplitude; they may reinforce each other or induce irregular structural responses. Design must therefore consider mechanical degrees of freedom, damping regulation, and power-management strategies to avoid disorderly impacts, structural fatigue, or unstable electrical output caused by multi-source excitation.

#### 2.2.3. Moisture-Resistant and Anti-Corrosion Configurations

Moisture resistance and corrosion protection are prerequisites for the long-term offshore operation of wind-driven TENGs [49]. High relative humidity promotes the adsorption of water molecules on triboelectric surfaces, which can screen surface charges and accelerate charge dissipation [50]. Salt-containing aerosols introduce an additional degradation pathway because deposited ions increase surface conductivity and can corrode metallic electrodes, interconnects, bearings, and electrical contacts. Consequently, offshore protection must address not only the triboelectric interface but also electrodes, mechanical components, power-management electronics, and sealing interfaces.

Current protection strategies can be broadly divided into surface modification, conformal barrier coatings, and structural encapsulation. Hydrophobic or superhydrophobic surface treatments [51] can reduce water adsorption while preserving the mechanical compliance of exposed triboelectric layers. Their main limitation is that protection depends on the integrity of a relatively thin surface layer; repeated contact, abrasion, UV exposure, or contamination may progressively reduce hydrophobicity. Conformal polymer coatings and intrinsically moisture-resistant materials [52] provide a more continuous barrier for electrodes and electrical interconnects, but increased coating thickness can alter flexibility, interfacial capacitance, heat dissipation, or mechanical response. By contrast, fully enclosed or hermetic architectures physically isolate sensitive triboelectric and electronic components from the external environment [53]. Such approaches generally provide stronger protection but may increase device mass, fabrication complexity, and sealing requirements and can interfere with the mechanical degrees of freedom required for wind-energy harvesting.

Corrosion protection must similarly be considered at multiple levels. Corrosion-resistant electrodes and conductive components [54] can reduce electrochemical degradation, whereas dense encapsulation and protected interconnects limit direct exposure to salt-containing moisture. For floating or buoy-based systems, sealing interfaces, cable feedthroughs, bearings, and joints may become more critical failure locations than the triboelectric material itself. Anti-fouling or self-cleaning surfaces can further suppress deposition and biological attachment during prolonged marine exposure. Therefore, offshore reliability depends on a multilayer protection strategy rather than on a single hydrophobic coating.

A major challenge in comparing these approaches is the lack of standardized accelerated-aging protocols. “Waterproof”, “humidity-resistant”, and “marine-compatible” are often used to describe substantially different levels of evidence. Short-term water immersion, operation under elevated relative humidity, salt-fog exposure, and prolonged seawater aging probe different degradation mechanisms and should not be regarded as equivalent durability tests. Accelerated validation should report relative humidity, temperature, exposure duration, salt concentration, UV conditions, and mechanical cycling conditions, together with electrical output retention before, during, and after exposure. Where relevant, changes in contact angle, electrode resistance, leakage current, and mechanical integrity can provide complementary indicators of protection failure.

### 2.3. Low-Altitude Scenarios: Broadband Wind Adaptation and Lightweight Architectures

Low-altitude wind fields typically refer to atmospheric boundary-layer flows from near ground level up to heights on the order of 10–100 m. Compared with the more stable winds at higher altitudes, low-altitude winds exhibit frequent speed fluctuations, pronounced vertical wind shear, high turbulence intensity, and rapid changes in flow direction. For tethered unmanned aerial vehicles (UAV) platforms, low-altitude communication nodes, temporarily deployed monitoring devices, and suspended sensing systems, low-altitude wind serves as both a potential energy source and an uncertain disturbance during operation and deployment.

In this scenario, the advantages of TENGs are mainly reflected in lightweight construction, broadband response, and embeddability. Low-altitude platforms are highly sensitive to mass and volume, which makes conventional rotary turbines difficult to integrate into limited spaces. In contrast, TENGs can be implemented using flexible films, lightweight support frames, bioinspired blades, or coaxial contra-rotating architectures, enabling modular installation on platform surfaces, edges, or hanging components. Moreover, the unsteady nature of low-altitude winds challenges conventional turbines in maintaining stable operation. Because TENGs can respond through multiple modes—contact–separation, fluttering, rotation, or vibration—they are inherently more capable of harvesting intermittent energy across a broad wind-speed range.

Notably, the design objective for low-altitude TENGs is not simply to maximize peak power. Instead, it should balance broadband wind adaptability, fatigue resistance, and suppression of output fluctuations. Under high-speed gusts, stochastic turbulence, and complex load variations, structural robustness and circuit-level matching largely determine whether a system is viable for real deployment.

#### 2.3.1. Bioinspired Adaptive Configurations

**Figure 7:**
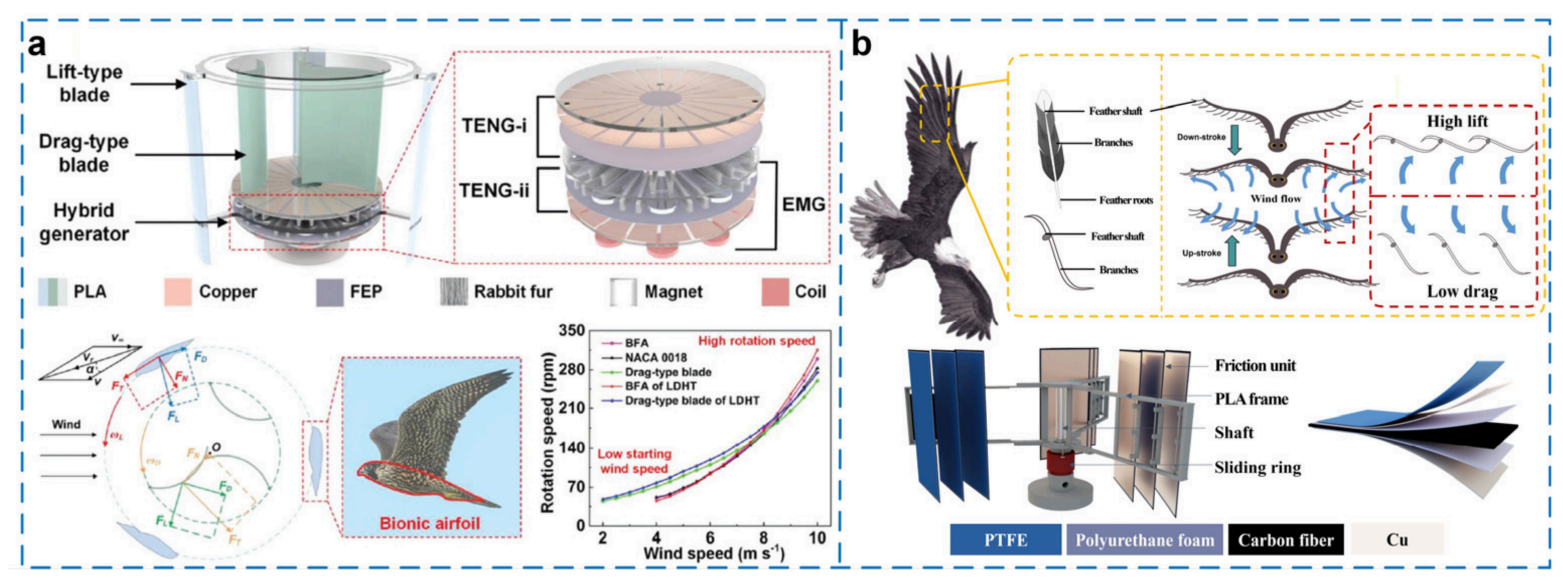
Bioinspired adaptive wind-driven TENGs: (**a**) Bionic blade lift–drag combined TENG with enhanced aerodynamic performance. Reproduced with permission from ref. [56]. Copyright (2023) John Wiley and Sons. (**b**) Feather-inspired TENG [57].

Bioinspired adaptive architectures mimic natural structures—such as plant leaves, bird wings, feathers, or organismal postures—to passively adapt to complex winds and improve aerodynamic performance [55,56,57,58]. Representative bioinspired designs include a lift–drag coupled bionic-blade TENG for enhanced aerodynamic energy capture (Figure 7a) and a feather-inspired TENG using aerodynamic lift and drag modulation (Figure 7b). These designs often exhibit low drag and fast response under weak winds, while under strong winds they can change shape and adjust the contact mode to avoid overload and fatigue damage. Compared with rigid structures, adaptive configurations provide better compatibility over a wide wind-speed range, enabling a more favorable trade-off between cut-in threshold and steady-state output.

The key challenge lies in balancing structural compliance and mechanical stability. Overly soft structures tend to produce unstable output, whereas overly stiff designs fail to respond effectively to low-speed airflow. Further advances in bioinspired adaptive TENGs therefore require co-optimization of material elasticity, structural geometry, and electrical output characteristics.

#### 2.3.2. Coaxial Contra-Rotating Configurations

**Figure 8:**
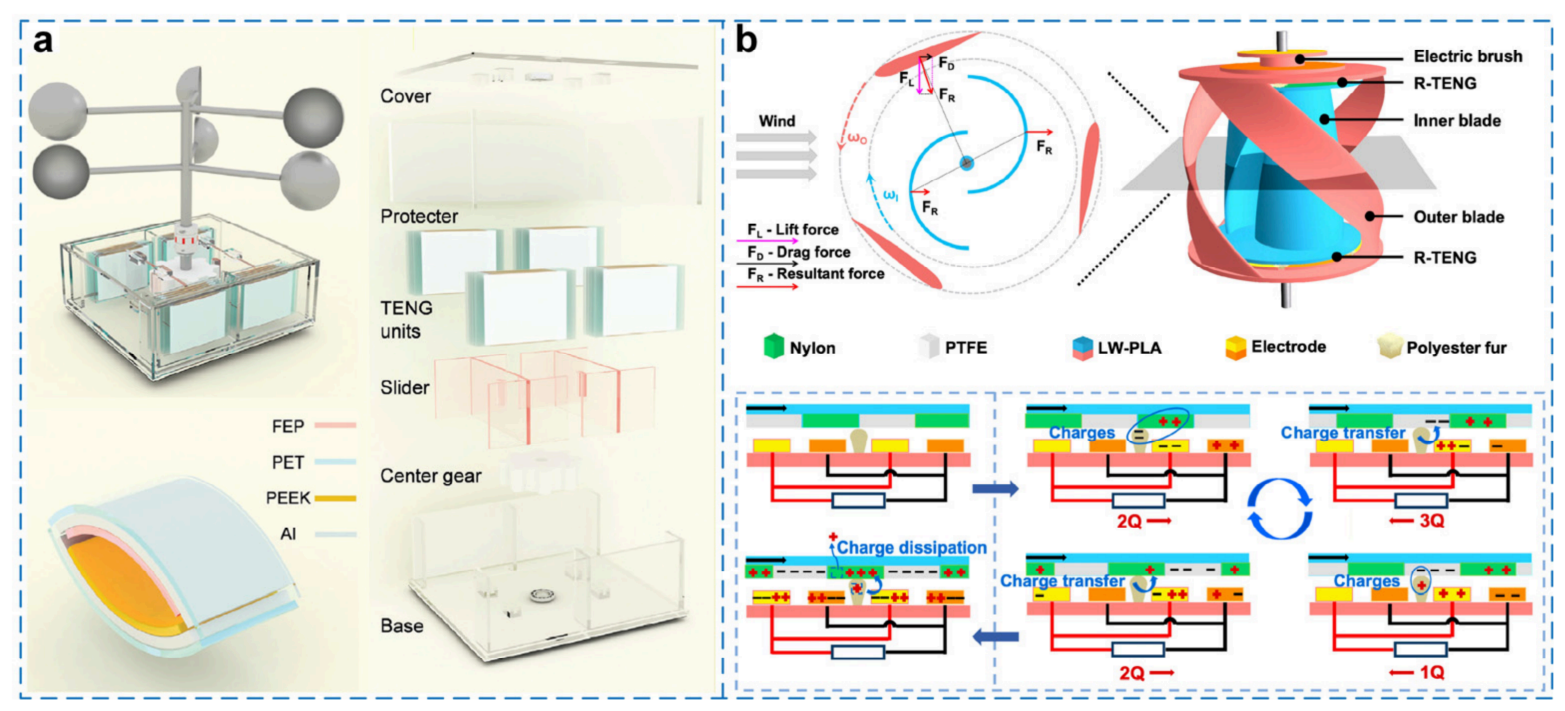
Coaxial contra-rotating wind-driven TENGs: (**a**) Bidirectional wind-cup-driven TENG. Reproduced with permission from ref. [61]. Copyright (2024) John Wiley and Sons. (**b**) Coaxial counter-rotating TENG driven by lift–drag hybrid blades [62].

Coaxial contra-rotating architectures employ two mechanical units rotating in opposite directions to improve torque balance and enhance output under complex wind conditions [59,60,61,62,63,64]. Representative rotary architectures include a bidirectional wind-cup-driven TENG (Figure 8a) and a coaxial counter-rotating TENG equipped with lift–drag hybrid blades (Figure 8b). A major advantage is the ability to broaden the operational wind-speed range while maintaining structural stability at higher wind speeds. For low-altitude platforms, contra-rotation can not only improve energy capture but also reduce vibration transmission to the host platform, thereby enhancing overall system stability.

In addition, these configurations are well suited to modular design and compact spatial integration, making them attractive for lightweight, detachable deployments. They are particularly relevant to low-altitude communication nodes and tethered monitoring platforms where engineering feasibility and ease of integration are crucial.

#### 2.3.3. Broadband Response and Lightweight Integration Strategies

Beyond coping with highly variable winds, low-altitude TENGs must be efficiently co-designed with their host platforms. Consequently, broadband response and lightweight integration have become key objectives. Broadband response refers to the capability to operate over a wider range of wind speeds and excitation frequencies [65,66], whereas lightweight integration requires deployment without imposing substantial mass penalties on the platform [67,68]. To this end, studies commonly adopt thin-film designs [69], compliant supports [70], foldable structures [71], and multi-unit modular arrays [72] to enhance overall adaptability.

In summary, architectural evolution in low-altitude scenarios is dominated by two keywords—broad adaptability and lightweight design—aiming to maintain sufficient stability and deployment flexibility in complex boundary-layer winds.

### 2.4. Scenario-Specific Requirements and Cross-Cutting Challenges for Wind-Driven TENGs

Overall, urban, offshore, and low-altitude wind environments all belong to complex, distributed wind fields, yet they differ substantially in constraints and application goals. Consequently, the design requirements for wind-driven TENGs also diverge (as summarized in Table 1). In urban micro-wind scenarios, priorities include low cut-in wind speed, turbulence tolerance, and compact deployment, which align with powering building-mounted sensors, environmental monitoring nodes, and smart infrastructure. In offshore settings, the key requirements shift toward corrosion resistance, moisture and thermal tolerance, long service life, and multi-source synergistic harvesting, targeting self-powered systems for buoys, marine monitoring platforms, and ocean engineering structures. In low-altitude complex winds, the emphasis is placed on broadband wind-speed adaptation, lightweight integration, and high operational reliability, which are critical for low-altitude communication devices and distributed sensing nodes.

Therefore, an application-driven research strategy should move beyond device-mode taxonomies and focus on wind-field-constrained architecture matching, system integration, and engineering deployment. Only by establishing a unified “scenario–structure–performance–application” framework can the field clearly define the value boundary and development pathway of wind-driven TENGs in wind energy utilization.

Despite the diversity of architectures across wind scenarios, several common design principles can be identified. First, almost all wind-driven TENGs aim to reduce cut-in wind speed and improve responsiveness to low-energy inputs. Second, most designs attempt to mitigate wear and charge decay through structural optimization, material modification, and circuit co-design. Third, as research transitions from laboratory prototypes to real environments, maintainability, packaging reliability, and long-term stability are becoming dominant evaluation criteria, rather than instantaneous output alone.

Accordingly, the evolution of wind-driven TENG architectures should not be viewed as a simple accumulation of structural innovations. Instead, it represents a progressive optimization process centered on the chain of scenario constraints, structural response, and system stability. The most valuable designs are not those that achieve the best performance in a single controlled test, but those that can operate stably, repeatably, and deployably in realistic wind fields over long durations.

## 3. Materials, Interfaces and System Integration: From Unit Devices to Engineering-Grade Systems

Performance improvements in wind-driven TENGs do not rely solely on mechanical-architecture optimization. They also critically depend on the materials system, interfacial engineering, environmental protection, packaging, and system-level integration. From an engineering perspective, these factors constitute the enabling foundation for translating wind-driven TENGs from laboratory prototypes into field-deployable technologies.

### 3.1. Materials Systems, Interfacial Engineering, and Environmental Packaging

#### 3.1.1. Triboelectric Materials and Dielectric-Layer Optimization

The selection of triboelectric materials is one of the primary determinants of TENG output. In general, a larger difference in electron affinity between the two contacting materials leads to a higher surface charge density generated during contact electrification, thereby increasing the output voltage and transferred charge. Commonly used negative triboelectric materials include PTFE [73], FEP [74], PDMS [75], PVDF [76], and their composites [77]. Typical positive triboelectric materials include nylon [78], metal films [79], cellulose-based materials [80], and conductive polymers [81]. The specific material pairing sets the upper bound and stability of charge generation under different wind-field excitations.

In parallel, dielectric-layer enhancement has become an important materials strategy. Introducing high-permittivity fillers, polar functional groups, or nanoparticles into the matrix can increase charge storage capability and polarization response, thereby improving TENG output [82]. However, such approaches often involve two practical trade-offs. First, nonuniform filler dispersion can introduce local defects and degrade mechanical reliability. Second, excessive pursuit of polarization enhancement may increase stiffness, which can reduce sensitivity to weak winds. Therefore, materials optimization should target co-optimization among electrical properties, mechanical compliance, and environmental adaptability rather than maximizing a single metric.

For wind-driven TENGs, materials design should not focus only on higher instantaneous output; it must also account for flexibility, mechanical strength, wear resistance, and environmental stability [83]. For instance, in urban micro-wind and low-altitude complex wind fields, materials typically require high compliance to enable rapid response under weak airflow [84]. In offshore environments, moisture tolerance, salt spray resistance, and chemical stability become more critical [85]. In this sense, material selection for wind-driven TENGs is inherently a multi-objective trade-off rather than a simple “high charge density first” problem.

#### 3.1.2. Micro–Nano Structures and Surface Charge Regulation

Beyond intrinsic material properties, micro–nano structuring of the triboelectric surface is a key route to enhancing wind-driven TENG performance. Microstructures, nanoscale grooves, particulate asperities, and hierarchical roughness can increase the effective contact area, prolong contact duration, and improve local charge accumulation efficiency [86]. This strategy is particularly beneficial under low wind speeds and intermittent flows, where mechanical excitation is weak and interfacial design is needed to amplify limited contact–separation effects.

**Figure 9:**
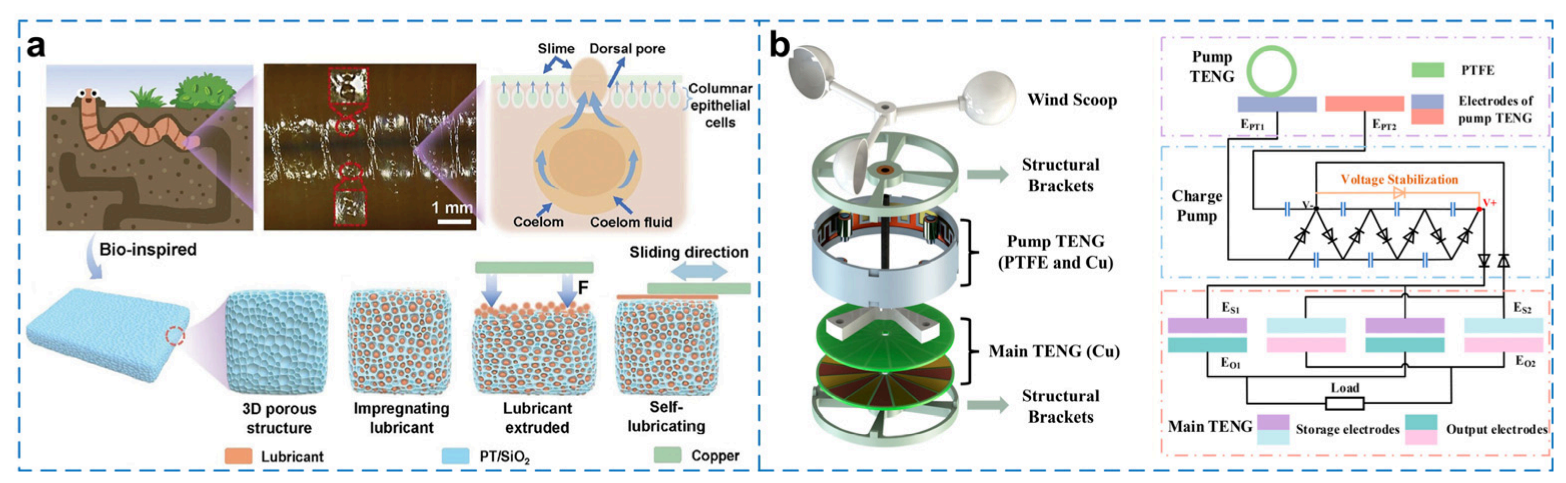
Micro–nano structure enhancement and surface charge regulation for wind-driven TENGs: (**a**) Bioinspired 3D porous network, self-lubricating wind-driven TENG [88]. (**b**) TENG with coaxial rolling charge pump strategy [89].

In wind-driven TENGs, surface-structure design must also align with the operating mode. For contact–separation and flutter-based devices, surface roughening can strengthen transient contact and local charge injection [87]. For sliding or rotary devices, interfacial design often prioritizes low friction, reduced wear, and stable charge transfer [88] (Figure 9a). Thus, micro–nano structures should be treated as mode-matched interfacial engineering rather than isolated surface modification.

Surface charge regulation provides another pathway to output enhancement. Representative strategies include charge pump circuits [89] (Figure 9b) and modified composite materials [90], which can improve surface charge density and charge retention to some extent. Importantly, higher initial surface charge does not necessarily translate into long-term stability. In realistic outdoor wind fields, charge decay is often governed by the combined effects of humidity, contamination, and wear. As a result, initial charge density alone is not a sufficient indicator of practical usability.

#### 3.1.3. Wear Resistance, Moisture Tolerance, and Anti-Fouling Design

A major barrier to real-world deployment of wind-driven TENGs is performance degradation in complex environments. In urban micro-wind scenarios, airborne dust, particulates, and pollutant deposition can accelerate tribolayer wear. Offshore, salt spray, persistently high humidity, and marine biofouling can severely compromise interfacial stability. For low-altitude platforms, frequent vibrations and gust-induced pulsations further amplify structural fatigue. Consequently, environmental adaptability has become a central design requirement rather than an optional add-on.

For wear mitigation, common approaches include non-contact or weak-contact operation modes [91], lubrication-assisted designs [92], high-toughness elastomers [93], and optimized motion trajectories [94] to reduce tribolayer damage. For humidity and salt spray resistance, superhydrophobic coatings [95] and dense encapsulation layers [96] are widely used to limit water-vapor and ion ingress. For anti-fouling, increasing attention has been paid to anti-bioadhesion surfaces [97] and self-cleaning interfaces [98], where engineered roughness and anti-fouling coatings can suppress biofilm formation and organic deposition.

A practical challenge remains: balancing long-term durability with environmental friendliness. Some high-performance coatings improve protection but introduce manufacturing complexity, higher cost, and end-of-life recycling difficulties. Hence, protection strategies suitable for wind-driven TENGs should ideally meet multiple constraints simultaneously, including long-term stability, low cost, and sustainability.

#### 3.1.4. Sustainable Materials and Environmentally Benign Packaging

As TENG deployment expands from laboratory settings to outdoor environments, sustainable materials and eco-friendly packaging are gaining importance. Wind-driven TENGs often rely on polymers, electrode films, flexible substrates, and composite coatings, some of which are difficult to degrade. After long-term use, these materials may contribute to microplastic pollution and raise disposal concerns [99]. Accordingly, biodegradable and recyclable materials are becoming an increasingly important research direction.

For example, bio-based materials [100], biodegradable polymers [101], and recyclable electrode materials [102] have been explored to develop more environmentally compatible TENG prototypes. These materials offer broad availability and good processability, and they can reduce the life-cycle environmental burden. However, compared with conventional polymers, sustainable materials may still face limitations in mechanical robustness, moisture resistance, and charge retention. Further optimization is therefore required for practical deployment.

Direct quantitative comparison between conventional fluoropolymers and sustainable alternatives requires caution. Reported surface charge density and electrical output depend not only on the intrinsic material but also on counter-material selection, surface roughness, contact pressure, operating frequency, humidity, device geometry, and charge-management strategy. Consequently, the output of two complete TENG devices should not be interpreted as a direct measurement of intrinsic material superiority. A more meaningful sustainability assessment should jointly consider electrical performance, output retention under humidity and wear, mechanical lifetime, material mass, recyclability, and end-of-life pathway.

The key engineering question is therefore not whether every biodegradable material can match the maximum charge density of PTFE or FEP, but whether it can deliver sufficient lifetime-integrated energy for a defined application with a lower environmental burden. For replaceable or relatively short-lifetime urban sensing nodes, moderate reductions in charge density may be acceptable if compensated through larger active area, surface functionalization, charge trapping, energy accumulation, or low-duty-cycle electronics. In contrast, offshore deployments impose stringent requirements on moisture resistance, mechanical lifetime, and long-term charge retention. In such environments, premature degradation of a nominally sustainable material may increase replacement frequency and life-cycle impact, potentially offsetting the environmental benefit of biodegradability.

Beyond material substitution, circular design provides another pathway toward sustainable TENG systems. Promising strategies include modular and replaceable triboelectric layers, design for disassembly, recyclable metal electrodes, reduction of permanent adhesive bonding, separation of electronic and triboelectric modules, recovery and reuse of mechanical frames, and standardized material labeling. Encapsulation should likewise be designed for replacement or recycling rather than permanently embedding all functional components in a non-separable polymer matrix. Future assessments should therefore move from a “biodegradable versus non-biodegradable” binary comparison toward life-cycle metrics that include material sourcing, fabrication energy, service lifetime, maintenance frequency, recovery efficiency, and environmental release during wear.

### 3.2. Impedance Matching and Power-Management Circuits

The development of wind-driven TENGs should not stop at improving generator-side output. The intrinsic electrical characteristics of TENGs—typically high source impedance, high voltage, low current, and pulsed or alternating waveforms—are poorly matched to most electronic loads [103]. Therefore, the engineering performance of a wind-driven TENG system depends critically on how efficiently its raw electrical output is converted into stored and regulated energy.

A complete power-management chain can be represented as TENG, rectification, impedance matching/charge extraction, intermediate storage, voltage regulation, load scheduling, and target load. Losses may occur at every stage. These losses are particularly important under weak or intermittent wind because the harvested average power may be comparable to the standby consumption of the power-management circuit itself.

Conventional full-wave bridge rectifiers provide a simple route for converting alternating TENG output into unidirectional charging current [104]. However, their suitability depends on the output amplitude, transferred charge, operating frequency, and effective source impedance. More advanced strategies, including synchronized or switched charge extraction, charge pumps, mechanical/electrical switching, and impedance-transforming interfaces [105,106,107,108], seek to increase the fraction of generated electrostatic energy transferred to storage. The benefit of these circuits should nevertheless be evaluated after subtracting their control and leakage losses rather than solely through idealized conversion ratios.

Energy storage acts as a temporal buffer between stochastic wind input and deterministic load demand. Capacitors and supercapacitors are attractive for frequent charge–discharge cycling and short-duration power bursts, whereas rechargeable batteries may provide greater energy capacity for longer duty cycles. The appropriate storage element depends on the statistical distribution of harvested energy, leakage characteristics, required output voltage, and load schedule. Thus, comparing charging curves requires reporting at least the capacitance or battery capacity, initial and final voltage, charging time, wind condition, and rectification/power-management topology.

Impedance matching for TENGs also differs from conventional low-impedance generators because the effective electrical behavior depends on capacitance, excitation frequency, and switching state. A fixed resistive load optimized under one wind speed may therefore be suboptimal under another. For realistic variable winds, adaptive or event-driven energy extraction is potentially more appropriate than optimization at a single laboratory operating point. However, increased circuit complexity is justified only if the additional harvested energy exceeds the associated control consumption and reliability penalty.

Power management must consequently be co-designed with both the wind field and target load. In urban micro-wind environments, ultra-low quiescent consumption and effective accumulation of sparse energy packets are primary requirements [109]. Offshore systems require not only efficient conversion but also robust encapsulation of power electronics and storage devices under high humidity and salt exposure [110]. Low-altitude platforms place additional constraints on circuit mass, volume, and response to rapid input fluctuations [111]. Across all scenarios, reporting regulated load-side energy rather than only raw TENG output will provide a more meaningful basis for engineering assessment.

### 3.3. System Integration

A single TENG, with its high-voltage and low-current output, cannot meet the diverse load requirements across scenarios—for example, urban sensors typically require stable low power, offshore buoys may need higher pulsed power, and low-altitude platforms demand broadband energy capture. As a result, hybrid integration of TENGs with other harvesting technologies—such as electromagnetic generators (EMGs), piezoelectric nanogenerators (PENGs), and photovoltaic cells (PVs)—has become a major trend. Such hybrid systems can exploit complementary strengths to cover broader wind-speed ranges and load profiles [112].

#### 3.3.1. Hybrid Integration of TENGs with EMGs/PENGs/PVs

Because a standalone TENG is limited in both output form and power scale, hybridization with other energy-harvesting technologies has been widely investigated.

**Figure 10:**
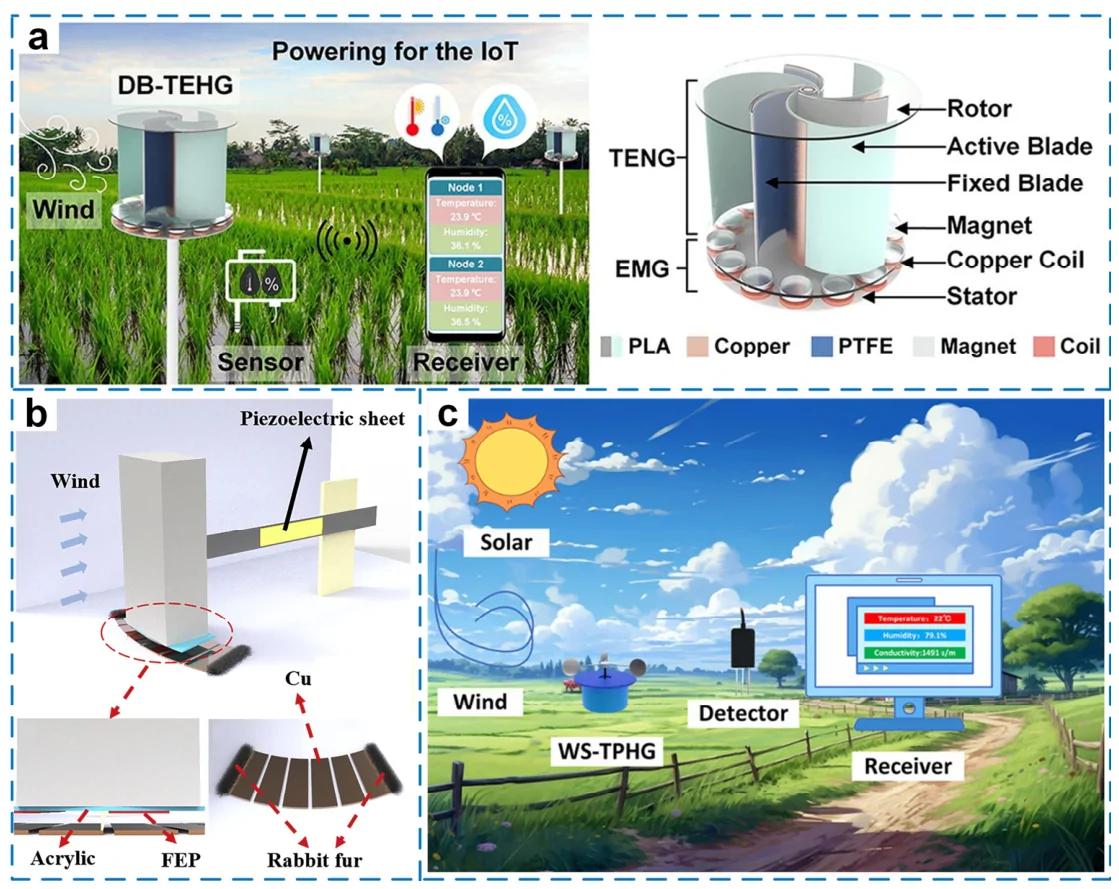
Hybrid energy harvesting systems integrating TENGs with other technologies: (**a**) Double-blade structured TEHG with aerodynamic enhancement for breeze energy harvesting. Reproduced with permission from ref. [113]. Copyright (2022) Elsevier. (**b**) Collision-free gallop-based TPHG [116]. (**c**) Hybrid wind–solar self-powered detector system using a TPVHG for smart agriculture. Reproduced with permission from ref. [119]. Copyright (2024) John Wiley and Sons.

Triboelectric–electromagnetic hybrid generators (TEHGs) are the most common for wind harvesting. The key idea is to combine the high-voltage output of TENGs with the high-current output of EMGs [113] (Figure 10a). In wind-energy systems, TENGs tend to be more responsive to low wind speeds and non-stationary excitations, whereas EMGs typically provide more stable current at higher rotational speeds [114]. Their coupling can therefore broaden the operational wind-speed range and improve output characteristics. However, hybridization is not a simple superposition. The two generators differ in optimal rotation speed, coupling frequency, volume allocation, and circuit requirements. Genuine performance gains require coordinated structural and circuit co-design [115]; otherwise, the system may become more complex without becoming more efficient.

Triboelectric–piezoelectric hybrid generators (TPHGs) often emphasize shared utilization of low-frequency vibration and elastic deformation [116] (Figure 10b). In scenarios such as wave-induced motion offshore, vibration on low-altitude platforms, or localized airflow disturbances around buildings, PENGs can serve as compliant supports or auxiliary transducers, improving broadband response to mechanical excitation [117]. These hybrids are particularly sensitive to small-amplitude, low-frequency motions, which is advantageous in complex and non-stationary environments. Nevertheless, because the transduction mechanisms differ substantially, synergy strongly depends on structural design and material coupling [118]. Poor coupling may even lead to mutual interference. Thus, the key to TPHG systems lies in coordination rather than mere combination.

Triboelectric–photovoltaic hybrid generators (TPVHGs) are practically appealing for offshore platforms, low-altitude monitoring nodes, and long-term deployments [119] (Figure 10c). Wind and solar resources often complement each other: PV can dominate under strong daylight, while TENGs can provide supplementary energy under cloudy conditions, at night, or under partial shading [120]. In regions with alternating wind and solar availability, such hybrid systems can improve supply continuity and robustness.

Overall, the primary benefit of hybrid energy systems is not only higher total output power but improved spatiotemporal continuity of the energy supply. For long-term, unattended monitoring nodes, supply stability is often more meaningful than peak power. Therefore, the synergy between TENGs and other harvesting technologies should be evaluated with an emphasis on system-level reliability rather than device-level maxima.

#### 3.3.2. Co-Design of Energy Storage, Sensing, and Loads

The output of wind-driven TENGs is typically pulsed and intermittent, which makes direct powering of electronics difficult. Hence, the co-design of energy storage, sensing, and loads is a necessary step toward engineering deployment. A complete wind-driven TENG system usually includes an energy-harvesting unit, rectification and power-management modules, an energy-storage unit, sensors, a controller, and communication modules [121]. Depending on application requirements, three representative coupling paradigms can be identified.

Direct-coupling mode. For ultra-low-power tasks, a TENG can drive micro-power sensors using only simple rectification and storage elements [122]. This mode is structurally simple, low cost, and straightforward to control, and it is suitable for intermittent monitoring tasks such as environmental parameter sampling, status indication, and short-burst wireless transmission [123]. However, it is highly sensitive to load power demand; even a modest increase in consumption can cause discontinuous operation.

Indirect-coupling mode. In more complex systems, the TENG output is first processed by a power-management circuit, and the load is powered from an energy-storage capacitor (or other storage unit) [124]. This mode is better suited to sensor nodes and wireless modules that require sustained operation [125]. Although it increases system complexity, it significantly improves supply stability and task continuity, making it more consistent with real deployment needs.

Self-powered sensing mode. In some scenarios, the TENG serves not only as an energy harvester but also as the sensing unit itself [126]. Because TENG outputs are highly sensitive to mechanical displacement, wind-speed variations, and vibration states, the electrical signal can directly encode environmental information. This mode integrates power generation and sensing, offering fast response, simplified structure, and high integration density [127]. A remaining challenge is signal decoupling: the output simultaneously reflects environmental stimuli, device dynamics, and loading conditions, which complicates calibration and robust interpretation in real wind fields.

### 3.4. System-Level Stability and Reliability

For wind-driven TENGs, system-level reliability is a decisive metric for real-world deployment. System stability encompasses mechanical, interfacial, circuit, and environmental stability. Mechanical stability concerns fatigue lifetime, structural integrity, and response consistency under long-term wind loading [128]. Interfacial stability focuses on tribolayer wear, charge decay, and surface contamination [129]. Circuit stability addresses the long-term robustness of rectification, energy storage, and power-management modules during continuous operation [130]. Environmental stability emphasizes device performance under harsh conditions such as high humidity, dust exposure, and temperature cycling [131].

From this perspective, research on wind-driven TENGs should move beyond demonstrating electricity generation and toward enabling continuous, stable, and deployable power delivery. The core engineering challenge is not whether a device can operate under ideal laboratory conditions, but whether it can sustain acceptable performance during long-term service in realistic environments.

## 4. Performance Evaluation, Engineering Applications, and Scenario-Oriented Deployment

Reports on wind-driven triboelectric nanogenerators (wind-driven TENGs) vary substantially across the literature. Even for the same device category, the reported output may differ by orders of magnitude due to differences in experimental platforms, wind-speed measurement methods, and the choice of load conditions, rectification circuits, and storage capacitors. This lack of comparability directly undermines the credibility of review-level conclusions and impedes engineering selection and cross-laboratory reproducibility. This issue is particularly important for a scenario-oriented assessment. Therefore, establishing and discussing a coherent performance-evaluation framework and identifying gaps in standardization are essential steps for moving from device demonstrations to deployable technologies [132].

Accordingly, this section establishes a quantitative evaluation framework for wind-driven TENGs. Representative devices are compared using reported aerodynamic, electrical, durability, and system-level parameters, while missing or inconsistently defined quantities are explicitly identified. Particular attention is given to three methodological issues that currently limit cross-study interpretation: the operational definition of cut-in wind speed, the normalization of output power, and the level of validation under realistic environmental conditions. The objective is not to construct a universal performance ranking from fundamentally heterogeneous experiments, but to determine which comparisons are technically defensible and which reporting gaps must be resolved before engineering selection becomes possible.

### 4.1. Performance Metrics and Operational Definitions

A meaningful assessment of wind-driven TENGs requires clear definitions of both the input excitation and the electrical/system response. For engineering purposes, the evaluation boundary should extend from the incoming wind field to the energy ultimately available to a target load. The relevant metrics can therefore be organized into wind-field characterization, generator-side electrical output, energy-conversion performance, and usable power-supply capability.

#### 4.1.1. Characterization of Wind-Field Inputs

Mean wind speed alone is insufficient to characterize the aerodynamic input of a wind-driven TENG. At a minimum, the reported wind condition should include the wind-speed range, measurement position relative to the device, wind-source type, device orientation, and, where relevant, wind-direction variation and turbulence intensity [133]. For devices relying on vortex-induced vibration, flutter, galloping, or rotational motion, the mechanically effective excitation should also be characterized through quantities such as oscillation frequency, rotational speed, vibration amplitude, vortex-shedding frequency, or reduced velocity.

This distinction is important because nominally identical wind speeds can produce markedly different mechanical responses depending on the flow profile and experimental configuration. For example, the velocity measured near the outlet of a laboratory fan may not represent the spatially averaged velocity incident on the full projected area of the harvester. Similarly, a nominal mean speed in a turbulent wake does not describe the transient aerodynamic loading experienced by a fluttering film. Where possible, turbulence intensity should therefore be reported together with mean wind speed.

For outdoor measurements, additional information such as sampling duration, averaging interval, wind-direction distribution, temperature, relative humidity, and installation height can substantially improve reproducibility. These parameters become especially important when laboratory and field performances are compared.

#### 4.1.2. Electrical Output and Usable-Energy Metrics

Generator-side characterization commonly includes open-circuit voltage (*V*_oc_), short-circuit current (*I*_sc_), transferred charge, load-dependent voltage/current, and maximum output power [134]. These quantities remain useful for characterizing electromechanical behavior, but they should not be treated as equivalent measures of useful energy.

Power density also requires careful interpretation. At least three normalization areas appear in the wind-energy-harvesting literature: the active triboelectric contact area, the projected wind-facing/interception area, and the total device footprint [135]. These quantities represent different physical boundaries and can produce substantially different numerical power densities. Therefore, every normalized performance value should identify its denominator explicitly. For wind-energy comparisons, projected wind-facing area is particularly relevant to aerodynamic resource utilization, whereas triboelectric active area may be more appropriate for assessing interfacial device performance. Neither should be reported simply as “area” without definition.

For practical power supply, average harvested energy and load-side performance are more informative than isolated peak values. Recommended system-level metrics include energy accumulated in a storage element per unit time, capacitor/battery charging rate, regulated output voltage, voltage ripple, supported load power, operating duty cycle, and the number or frequency of sensing/communication events that can be sustained [136]. When power-management circuitry is used, the measurement boundary should also specify whether the reported value corresponds to the raw TENG terminals, the rectifier output, the storage element, or the regulated load.

A useful distinction can therefore be made between generator-side performance and usable-energy performance. The former characterizes the transducer itself, whereas the latter determines whether a complete harvesting system can satisfy a specified application. This distinction is central to avoiding overinterpretation of high instantaneous voltage or peak power as evidence of autonomous power-supply capability.

#### 4.1.3. Operational Definition of Cut-In Wind Speed

Low cut-in wind speed is frequently cited as a principal advantage of wind-driven TENGs. However, the term “cut-in wind speed” is not used consistently across the literature. Depending on the experimental criterion, it may refer to the onset of mechanical motion, the first detectable electrical signal, or the onset of practically usable energy generation. These thresholds are physically distinct and should not be used interchangeably.

For clarity, three operational thresholds can be distinguished:

(1) Mechanical onset speed (*v*_mech_): The minimum wind speed at which the intended sustained mechanical response—such as flutter, galloping, vortex-induced vibration, or continuous rotation—is initiated.

(2) Electrical detection threshold (*v*_det_): The minimum wind speed at which the TENG produces an electrical signal that can be distinguished from the instrumental noise floor under a specified measurement configuration.

(3) Usable-power threshold (*v*_use_): The minimum wind speed at which the complete energy-harvesting system, after rectification, energy extraction, storage, and/or voltage regulation, can accumulate or deliver sufficient energy to satisfy a predefined target load or operating duty cycle.

These three thresholds need not coincide. A flexible fluttering device may begin oscillating before sufficient contact or charge transfer occurs to produce a robust electrical signal. Conversely, a high-impedance measurement instrument may detect voltage pulses at a very low wind speed even though the associated harvested energy is insufficient to charge a storage capacitor or operate an electronic load. In practical systems, *v*_use_ is therefore generally the most application-relevant threshold.

This distinction also affects interpretation of the literature values. When an original publication experimentally determines an onset wind speed using a clearly stated criterion, the value can reasonably be reported as a cut-in threshold together with that criterion. When a study merely demonstrates operation at its lowest tested wind speed, however, the value should be described as the “minimum tested operating wind speed” rather than the actual cut-in wind speed. The true threshold may lie below—or, depending on the criterion for usable power, effectively above—that test condition.

Accordingly, future studies should avoid reporting “cut-in wind speed” without an explicit operational definition. For engineering comparison, reporting both *v*_det_ and *v*_use_ would be particularly valuable because it separates device sensitivity from practical power-supply capability. Thus, a low electrical detection threshold should not by itself be interpreted as evidence of useful low-wind power generation.

### 4.2. Field-Validation Maturity in Realistic Wind Environments

Because realistic wind environments form the central scope of this review, it is necessary to distinguish between a device proposed for a real-world application and a device actually validated under representative field conditions. Application-oriented schematics or demonstrations under a laboratory fan do not by themselves establish environmental readiness. We therefore classify validation evidence into four levels according to the highest experimental condition demonstrated.

Level I—Controlled laboratory wind validation. The device is tested using a laboratory fan or wind tunnel under prescribed wind speeds, typically with limited environmental variation. Such tests are essential for mechanism characterization and repeatable comparison but provide limited evidence of resistance to realistic environmental stressors.

Level II—Environmentally or aerodynamically representative laboratory validation. In addition to controlled airflow, the device is exposed to one or more representative disturbances or environmental stressors, such as variable wind direction, turbulence, high humidity, water exposure, salt fog, dust, temperature variation, or irregular mechanical excitation. These experiments improve environmental relevance but generally remain accelerated or simplified representations of field conditions.

Level III—Short-term field or on-site demonstration. The harvester operates in an actual target or analogous environment—for example, on a building, bridge, traffic infrastructure, marine platform, agricultural site, or low-altitude platform—for a limited observation period. Such demonstrations verify integration feasibility and response to naturally varying inputs but may not provide sufficient duration to assess lifetime degradation.

Level IV—Long-term field validation. The complete harvesting system is operated for an extended period under actual environmental exposure, preferably with concurrent records of wind conditions, environmental variables, load operation, output retention, failure events, and maintenance interventions. This level provides the strongest evidence relevant to deployability and lifetime prediction.

This framework is intended as a validation-evidence classification rather than a formal technology-readiness-level assessment. Representative studies are assigned according to experimentally demonstrated validation, rather than the application scenario claimed in the publication.

The literature reviewed here indicates an important maturity gap. Although numerous wind-driven TENGs are motivated by urban, offshore, or low-altitude applications, a large fraction of reported performance remains derived from Level I laboratory tests. Level II studies increasingly address individual factors such as humidity, water exposure, or irregular wind, and Level III demonstrations are emerging for sensing and monitoring applications. In contrast, Level IV evidence combining prolonged natural wind exposure, environmental monitoring, actual load operation, and maintenance/degradation records remains limited. Therefore, “realistic-environment compatibility” should presently be interpreted primarily as a design objective for much of the field rather than as universally established field readiness.

### 4.3. Cross-Study Comparability and Reporting Completeness

The quantitative availability of a performance value does not necessarily make it suitable for cross-study comparison. Meaningful comparison requires sufficiently consistent boundaries for the aerodynamic input, device geometry, electrical output, and data-processing method. To evaluate this issue explicitly, the representative studies can be further assessed according to six reporting dimensions: (i) aerodynamic input characterization; (ii) device geometry and normalization area; (iii) electrical load condition; (iv) temporal definition of power; (v) power-management and storage boundary; and (vi) environmental/durability condition.

Aerodynamic input is considered adequately characterized when the wind source, velocity range, measurement location, and relevant device orientation are provided; turbulence intensity or temporal wind statistics provide a higher level of characterization for non-steady flows. Geometric reporting requires sufficient dimensions to identify the active and/or projected wind-facing area. Electrical reporting requires a defined load when power is quoted, while the distinction between instantaneous peak, cycle-averaged, and time-averaged power should be clear. At the system level, rectification and storage conditions must be specified if charging performance is used as evidence of practical output.

Based on these criteria, cross-study evidence can be divided into three practical comparability classes. Class A studies provide sufficiently defined aerodynamic, geometric, and electrical boundaries to permit approximate quantitative comparison with studies using similar definitions. Class B studies provide useful performance data but omit at least one major normalization or system boundary, restricting comparison to selected parameters. Class C studies primarily demonstrate the operating principle or report quantities such as *V*_oc_ and *I*_sc_ without sufficient information for meaningful power or efficiency comparison. This classification is not intended to evaluate the scientific quality of individual studies. Rather, it reflects whether the reported information is sufficient for secondary quantitative analysis and engineering selection.

### 4.4. Quantitative Benchmarking of Representative Wind-Driven TENGs

To move beyond qualitative architecture classification, Table 2 compares representative wind-driven TENGs across the major device categories and application scenarios discussed in this review. The selected parameters include wind-speed range, reported cut-in or minimum tested operating wind speed, device dimensions or projected wind-facing area, *V*_oc_, *I*_sc_, matched load, maximum and/or average output power, reported power density, capacitor-charging behavior, durability, and validation environment.

The table is intended as an engineering benchmark rather than a performance ranking. Values are retained as reported in the original publications unless an unambiguous conversion of units is possible. In particular, power densities are not re-normalized when the source study does not provide sufficient geometric information to reconstruct a common reference area. Similarly, a minimum tested wind speed is not reclassified as a cut-in speed unless the original study explicitly establishes an onset criterion. Missing quantities are marked as “NR” (not reported).

Several general observations emerge from this comparison. First, device categories differ not only in maximum output but also in the nature of their activation mechanism. Flutter- and flag-based systems can exhibit mechanical response under relatively weak airflow, whereas rotary devices may trade a higher mechanical activation requirement for more regular periodic output. Vortex-induced systems depend strongly on bluff-body geometry and flow-regime matching, while bioinspired adaptive structures seek to broaden the operating envelope through passive aerodynamic reconfiguration. Consequently, comparing only the lowest reported wind speed can obscure substantial differences in usable power and operating bandwidth.

Second, high reported voltage does not necessarily correspond to high average energy delivery. This is particularly important for TENGs because their large source impedance allows high open-circuit voltages to coexist with small charge transfer and limited current. Studies that additionally report matched-load power, capacitor charging, or operation of a real load therefore provide substantially stronger evidence of engineering capability than studies reporting *V*_oc_ and *I*_sc_ alone.

Third, durability data remain highly heterogeneous. Cycle counts under controlled laboratory excitation, continuous operating time in a wind tunnel, environmental aging tests, and outdoor exposure represent different forms of reliability evidence and should not be interpreted as interchangeable lifetime metrics.

Fourth, the apparent performance hierarchy changes depending on the metric selected. Architectures with the lowest reported activation thresholds do not necessarily exhibit the highest load-side average power, while devices with high peak output are not necessarily those with the strongest environmental validation. Moreover, missing projected-area definitions and inconsistent treatment of peak versus average power prevent rigorous power-density normalization for several otherwise representative studies. These results reinforce the need to separate device sensitivity, generator performance, usable energy, and field readiness as distinct assessment dimensions.

### 4.5. From Generator-Side Output to Practical Power Supply

For deployment-oriented evaluation, the relevant electrical boundary should extend beyond the TENG terminals. A typical energy pathway comprises the TENG transducer, rectification, impedance matching or charge extraction, intermediate storage, voltage regulation, and finally the target load. Each stage introduces losses and operational constraints, making load-side energy availability generally lower than generator-side matched-load output.

Rectification is particularly important because TENGs typically produce alternating or pulsed waveforms with high source impedance. Conventional diode bridges may introduce undesirable losses under low-current operation, while specialized switching, charge-extraction, or voltage-management strategies can improve energy transfer. However, the additional control circuitry may itself consume quiescent power. The net benefit must therefore be evaluated over the complete operating cycle rather than inferred from instantaneous conversion efficiency.

Storage introduces another important boundary. A capacitor-charging experiment provides stronger system-level information than Voc alone because the stored energy can be calculated from the capacitance and voltage change. Nevertheless, charging curves are comparable only when capacitor value, initial/final voltage, charging duration, rectification topology, and wind conditions are all reported. Demonstrating that a capacitor reaches a particular voltage without specifying these conditions provides limited quantitative information.

Ultimately, the most deployment-relevant metric is the energy delivered to a defined load over time. For intermittent sensing nodes, this may be expressed as the sustainable number of sensing-and-transmission cycles per hour or day. For continuously operating electronics, average regulated load power and voltage stability are more appropriate. Accordingly, future studies should report the energy flow across the complete chain wherever possible: wind/mechanical input, TENG, rectification, storage, regulation and load.

When the mechanical input can be quantified reliably, end-to-end energy-conversion efficiency can be defined relative to that input. When it cannot, power-management efficiency should instead be reported using clearly identified electrical input and output boundaries. These two definitions should not be conflated. In either case, generator-side peak power and load-side usable power should be reported separately.

### 4.6. Engineering Applications and Scenario-Oriented Deployment

The core engineering value of wind-driven TENGs is not to compete with conventional wind turbines for grid-scale power generation, but to provide in situ energy support for low-power, distributed, and long-term unattended terminals. For applications such as urban building monitoring, marine environmental observation, low-altitude platform power supply, and IoT sensor networks, wind-driven TENGs are attractive due to flexible installation, low maintenance cost, and deep integrability with existing infrastructure [137]. Accordingly, the focus of engineering discussion should shift from “whether the device can generate electricity” to “whether the system can stably power a target load over long durations”.

#### 4.6.1. Urban Micro-Energy Systems

**Figure 11:**
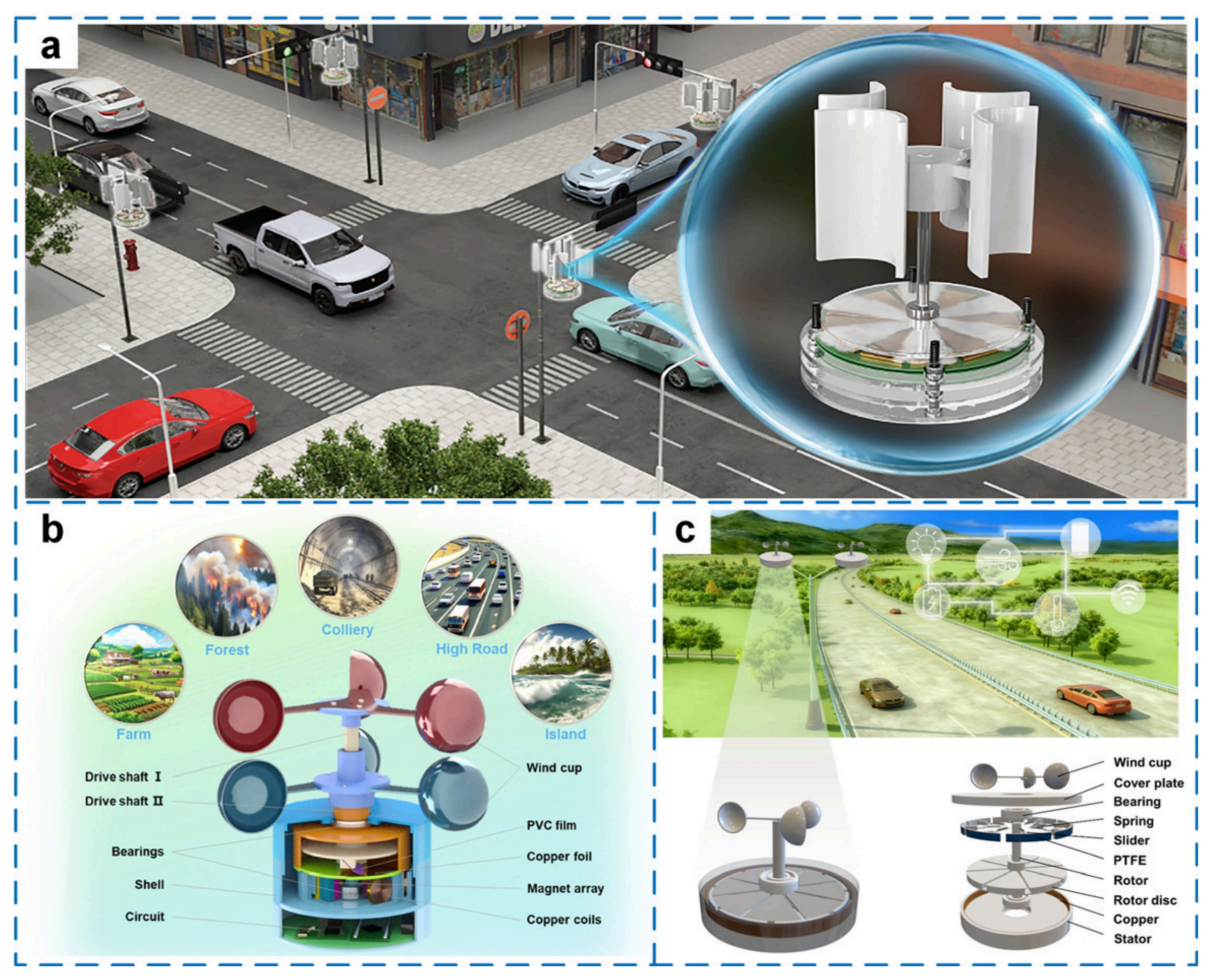
Wind-driven TENG deployment in urban micro-energy systems: (**a**) Wind-driven TENG in urban traffic environments. Reproduced with permission from ref. [141]. Copyright (2024) John Wiley and Sons. (**b**) All-in-one self-powered wind-speed sensor based on TENG [142]. (**c**) Wind-driven TENG for self-powered wireless sensing [144].

Urban micro-energy systems represent one of the most representative application directions for wind-driven TENGs (Figure 11). Cities contain abundant localized micro-wind resources that are difficult for conventional turbines to exploit, such as building rooftops [138], bridges [139], tunnels [140], and areas around transportation infrastructure [141] (Figure 11a). Although wind speeds are often low, these locations may exhibit locally accelerated airflow, high spatial density of available installation sites, and strong compatibility with infrastructure embedding conditions well aligned with small-scale, flexible, and distributed TENG deployment.

In this context, wind-driven TENGs are typically not intended to deliver high power. Instead, they target micro-power tasks such as environmental monitoring [142] (Figure 11b), SHM [143], and continuous powering of wireless sensor nodes [144] (Figure 11c). In smart-building and smart-city deployments, large numbers of air-quality, temperature and humidity, vibration, and occupancy sensors are distributed across indoor and outdoor spaces. Their individual power demands are low, but the overall network is extensive, making distributed micro-energy supply a natural fit [145].

From a deployment standpoint, urban applications impose three main requirements: (i) low cut-in wind speed to respond to weak or intermittent airflow; (ii) minimal noise and noticeable mechanical vibration to satisfy urban constraints; and (iii) good aesthetic integration and installation flexibility to enable embedding into existing buildings and facilities. These requirements favor “invisible integration” and low-maintenance operation rather than turbine-like external installations.

Urban scenarios also involve clear challenges: highly unsteady winds cause strong output fluctuations; airborne dust and pollutants accelerate contamination and wear; and building constraints raise the bar for device size, form factor, and safety. Therefore, urban micro-energy systems are generally better served by modular, small-area, multi-point deployment strategies rather than pursuing high power from a single site.

#### 4.6.2. Self-Powered Offshore Buoys and Monitoring Platforms

Offshore environments are another major application domain for wind-driven TENGs. Compared with urban scenarios, marine settings often provide more persistent wind resources, but they also introduce high humidity, salt spray corrosion, biofouling, and long-term unattended operation. For buoys, offshore monitoring stations, nearshore platforms, and SHM of marine infrastructure, the energy system must deliver not only electrical output but also strong environmental robustness and long-term reliability.

**Figure 12:**
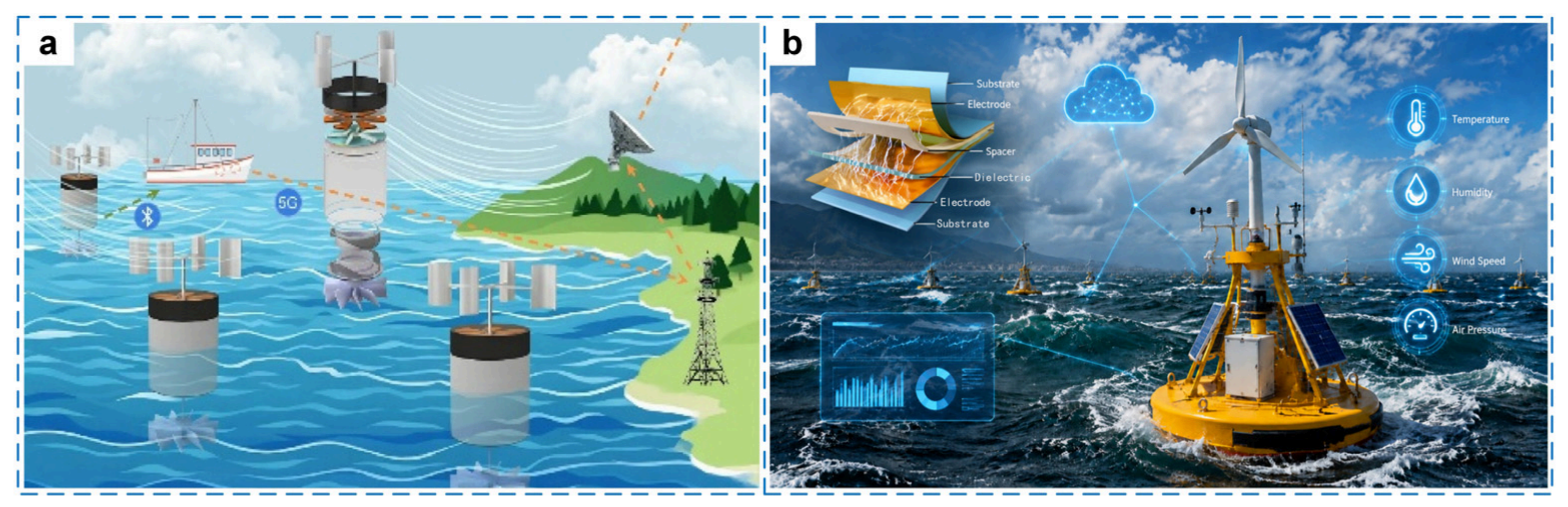
Wind-driven TENG deployment in marine environments: (**a**) Hybrid TENG for harvesting wind and water-flow energy [148]. (**b**) Schematic diagram of wind-driven TENG coupled with offshore buoy applied to self-powered monitoring.

A key advantage of wind-driven TENGs offshore lies in multi-source synergy [146]. Marine platforms are simultaneously subject to wind, waves, and platform motion. TENGs can therefore implement wind–wave synergy [147,148] (Figure 12a) or buoy-coupled harvesting [149,150] (Figure 12b) to improve continuity and stability. For long-term buoy systems, this multi-source capability is often more valuable than a high peak output, because onboard sensors and communication modules require stable, predictable, and sustainable low-power supply.

At the same time, offshore deployment places stringent requirements on packaging and materials. Salt spray and humidity accelerate electrode corrosion and interfacial degradation, while biofouling can further reduce performance. Thus, durability-oriented materials, anti-corrosion packaging, anti-fouling design, and system-level sealing must be treated as core design elements rather than auxiliary considerations. Ultimately, success offshore depends less on peak power and more on sustained operation under harsh conditions. To enable scale-up, long-term outdoor validation, accelerated marine aging protocols, and maintenance-cost assessment frameworks are necessary.

#### 4.6.3. Low-Altitude Platforms and Distributed Sensing Networks

Low-altitude scenarios have emerged as an increasingly active direction in recent years, including UAVs [151], tethered balloons [152], smart agriculture [153], and distributed sensing networks [154]. These platforms typically impose strict constraints on mass, volume, and integration density, while operating under strongly fluctuating winds. Such requirements favor lightweight and flexible wind-driven TENGs.

From an application perspective, three issues are central: (i) lightweight design to avoid increasing payload; (ii) broad adaptability to rapid changes in wind speed and direction; and (iii) compatibility with existing power-management, sensing, and communication modules. Rather than maximizing absolute output, low-altitude applications prioritize specific power (per unit mass), operational stability, and minimal interference with platform dynamics.

Practical challenges also exist. Devices may suffer fatigue damage under strong winds or turbulence; wind conditions vary significantly with location and altitude, reducing repeatability; and integrating harvesting, storage, and communication modules within limited space remains difficult. Hence, low-altitude deployment often favors small, modular, and detachable TENG solutions instead of large, highly coupled assemblies.

#### 4.6.4. Applicability Boundaries and Application Positioning

Across these scenarios, the applicability boundary of wind-driven TENGs is relatively clear: they are not intended for large-scale, grid-connected power generation requiring continuous high output. Instead, they are better positioned as in situ micro-energy units for low-power devices—particularly in distributed, hard-to-wire, and long-term unattended settings. Multi-source synergy and load-adaptive design can further improve usability under realistic conditions [155]. In other words, the central positioning of wind-driven TENGs is to provide localized energy support for end devices that are low-power but widely dispersed.

This positioning also implies that evaluation criteria must be adjusted accordingly. For urban, offshore, and low-altitude scenarios, comparing peak power alone is insufficient. More relevant metrics include power-supply stability in real environments, ease of deployment, long-term reliability, and operation cost. Only when application positioning and evaluation frameworks evolve in parallel can laboratory prototypes be translated into engineering-ready technologies.

To clarify this application boundary, Table 3 provides an application-oriented comparison between conventional wind turbines and wind-driven TENGs. Importantly, the comparison distinguishes system-level characteristics of conventional wind-energy systems from device-level electrical characteristics of TENGs and avoids treating the two technologies as direct competitors. Conventional wind turbines are mature technologies for substantial electrical power generation, whereas wind-driven TENGs are currently better considered as complementary micro-energy harvesters for distributed low-power applications.

Accordingly, wind-driven TENGs and conventional wind turbines should be regarded as technologies addressing different segments of the wind-energy landscape. Their most meaningful comparison is not which technology produces more power, but which better satisfies the energy, space, maintenance, environmental, and deployment constraints of a specific application.

### 4.7. Cost, Manufacturability, and Deployment Economics

Cost and manufacturability are important considerations for the practical deployment of wind-driven TENGs, yet quantitative economic data remain scarce. Most studies emphasize device-level performance, while material consumption, fabrication yield, packaging, installation, and maintenance costs are rarely reported. Consequently, reliable cross-study comparisons based on cost per watt are currently difficult.

At the device level, commonly used polymers such as PTFE, FEP, PVDF, and PDMS are compatible with established processing techniques, while Al and Cu electrodes benefit from mature manufacturing routes. However, micro/nanostructuring, surface functionalization, multilayer fabrication, and specialized coatings can substantially increase manufacturing complexity. Architecture is another cost driver: flutter- and flag-type TENGs generally employ relatively simple flexible components, whereas rotary, differential, and hybrid systems may require bearings, transmission components, magnets, coils, and precision assembly.

At the system level, the triboelectric element represents only part of the total deployment cost. Power-management circuits, energy storage, environmental packaging, mounting structures, and maintenance must also be considered. These costs are scenario-dependent: urban deployments emphasize scalable installation, offshore systems require corrosion-resistant packaging and costly maintenance access, and low-altitude platforms impose additional requirements for lightweight integration. Moreover, maintenance-free operation should not be assumed because tribolayer wear, contamination, encapsulation aging, and storage/electronic-component degradation remain unresolved concerns.

Conventional cost-per-watt metrics may therefore be insufficient for TENGs, whose primary role is distributed micro-energy supply rather than bulk electricity generation. For autonomous sensor nodes, more relevant indicators may include lifetime-delivered energy per unit cost, cost per powered node, maintenance interval, and avoided battery-replacement or wiring cost. Future studies should report bill-of-material estimates, manufacturing complexity, expected service life, and maintenance requirements alongside electrical performance to enable meaningful techno-economic assessment.

## 5. Critical Analysis and Outlook

Although wind-driven TENGs have demonstrated strong scenario compatibility in urban micro-winds, offshore wind fields, and low-altitude complex flows, the transition from laboratory prototypes to engineering deployment is still constrained by systemic bottlenecks. These barriers cannot be reduced to “insufficient output.” More fundamentally, a gap remains among evaluation methodology, engineering usability, and reliability assurance.

### 5.1. Key Challenges and Technical Bottlenecks

#### 5.1.1. Long-Term Stability and Lifetime Prediction: A Lack of Engineering-Decision-Ready Models

Long-term stability is arguably the most critical—and most underestimated—issue for wind-driven TENGs. Many studies demonstrate durability through thousands to hundreds of thousands of cycles. However, real wind-field operation exposes devices to coupled stressors, including humidity, dust, UV radiation, temperature fluctuations, salt spray, and random impacts. Laboratory cycling tests rarely reproduce the combined effects that drive interfacial aging and structural fatigue in outdoor service. Moreover, aging of encapsulation materials, stability of protective (and potentially sustainable) coatings, and maintenance strategies (e.g., replaceable tribolayers) must also be incorporated into lifetime assessments.

A more fundamental limitation is that many reports stop at a binary statement of whether output decreases after a given number of cycles, while lacking: (i) degradation-kinetics models relevant to realistic wind fields; (ii) lifetime prediction methods that take wind-speed distributions, turbulence intensity, humidity, and contamination as inputs; and (iii) an operational definition of “useful life” that is explicitly linked to maintenance strategy. As a result, engineering stakeholders cannot translate “laboratory lifetime” into deployable lifetime, maintenance intervals, or life-cycle cost.

#### 5.1.2. Lack of Standardization: Distorted Cross-Study Comparisons and Impeded Industrial Selection

The absence of standardized protocols spans input conditions, test methods, data processing, and reporting formats. Variations in wind-tunnel dimensions, wind-speed ranges, load resistance, humidity conditions, and device size make it difficult to compare voltage, power density, and efficiency across studies. In addition, some works report only peak outputs without average power, storage-charging curves, or load runtime, which can systematically overestimate practical performance.

This non-uniformity has two direct consequences. First, reviewers and researchers cannot reliably rank performance or identify the most promising technical routes. Second, industrial users cannot make selection decisions or estimate verification cost based on literature data. Standardization should therefore be framed not as a “formal requirement,” but as a prerequisite for making engineering usability quantifiable and transferable.

#### 5.1.3. Environmental Adaptability and Sustainability: Devices Must Not Only Work, but Work Reliably over Time

Environmental adaptability is both a key advantage and a major risk source for wind-driven TENGs. High humidity, dust, temperature cycling, and UV exposure directly affect charge retention and mechanical lifetime at triboelectric interfaces. Meanwhile, fluorinated polymers, nanofillers, and non-degradable encapsulation materials raise sustainability concerns. Future evaluation frameworks should move beyond output metrics to include material sourcing, life-cycle impacts, recyclability, and environmental risk.

A critical observation is that many studies still prioritize “high performance” materials without systematic assessment of recyclability, disassemblability, and life-cycle carbon footprint. Such omissions may become limiting factors in large-scale deployment, particularly in regions with tightening environmental regulations.

#### 5.1.4. The Gap from “Power Generation Demonstration” to “Deployment Decision”

These bottlenecks can be summarized hierarchically: Device level: output characteristics and cut-in threshold; System level: storage–regulation–load supply capability; Reliability level: long-term degradation and maintainability; Engineering-decision level: standardized evaluation and predictive lifetime models.

The current literature is relatively mature at the first two levels but insufficient at the latter two, leading to a practical situation where wind-driven TENGs are “demonstrable but difficult to deploy.”

### 5.2. Future Directions and Research Roadmap

Despite substantial progress in device architecture, materials engineering, and system integration, the next stage of wind-driven TENG development requires a transition from broad research directions toward verifiable engineering targets. Based on the gaps identified above, five grand challenges are proposed: (i) reproducing and characterizing realistic wind inputs; (ii) converting low-speed response into usable regulated energy; (iii) achieving predictable lifetime under coupled environmental stressors; (iv) scaling from individual devices to modular and manufacturable arrays; and (v) establishing sustainable, standardized, and economically assessable deployment. For each challenge, progress should be evaluated using measurable milestones rather than isolated improvements in peak electrical output.

#### 5.2.1. Customized Designs for Realistic Wind Fields

Most wind-driven TENGs are optimized under controlled and approximately steady airflow, whereas urban, offshore, and low-altitude winds exhibit turbulence, directional variation, gusts, wind shear, and environmental coupling. The first grand challenge is therefore to establish a direct link between realistic wind statistics and device design rather than optimizing structures at a single nominal wind speed.

Near-term research should characterize not only mean wind speed but also turbulence intensity, directionality, fluctuation spectrum, and installation-dependent flow conditions. Device optimization should subsequently incorporate these parameters into structural, material, and circuit design. Scenario-specific testing should reproduce representative urban wakes, offshore wind–wave excitation, and low-altitude gust/shear conditions.

A practical milestone would be the transition from performance curves based solely on steady wind speed to multidimensional performance maps linking wind statistics to mechanical response, average harvested energy, and usable load power. Ultimately, scenario-customized TENGs should be validated using measured field wind distributions rather than idealized laboratory inputs.

#### 5.2.2. From Low-Speed Activation to Usable Regulated Energy

Low cut-in wind speed and high open-circuit voltage are frequently emphasized, but neither guarantees useful power delivery. The second grand challenge is to close the gap between generator-side response and energy available to a target load. Future studies should distinguish mechanical onset, electrical detection threshold, and usable-power threshold, while evaluating the complete chain of TENG, rectification/energy extraction, storage, regulation and load.

Near-term priorities include ultra-low-quiescent power-management circuits, adaptive impedance matching under variable wind speeds, and systematic reporting of storage and load-side energy. Hybridization with EMGs, PENGs, or PVs should be justified by improvements in net energy availability rather than by the simple addition of peak outputs.

Key milestones should include routine reporting of average regulated power, charging energy per unit time, power-management efficiency, and sustainable load duty cycles under specified wind conditions. A mature system should demonstrate autonomous sensing and communication over statistically representative wind profiles without relying on an externally powered control circuit.

#### 5.2.3. Theory and Methodology for Array-Scale and Modular Designs

Laboratory cycling tests alone cannot establish service lifetime because realistic deployment involves coupled mechanical and environmental degradation. The third grand challenge is to develop lifetime-prediction methodologies that connect accelerated testing with field degradation.

Near-term studies should identify dominant failure modes, including tribolayer wear, charge decay, moisture ingress, corrosion, UV aging, contamination, mechanical fatigue, and encapsulation failure. Accelerated tests should progressively move from single-stressor exposure toward combined humidity–temperature–UV–salt/dust–mechanical loading, with output retention and failure modes recorded using consistent criteria.

A key milestone is the establishment of degradation models that predict performance retention as a function of environmental and mechanical exposure rather than simply reporting the number of completed cycles. These models should ultimately be validated against long-term outdoor data and used to estimate service life, maintenance intervals, and replaceable-component schedules for specific deployment scenarios.

#### 5.2.4. Standardized Protocols and Evaluation Criteria

Increasing system output through arraying is not equivalent to multiplying the output of an isolated TENG. Aerodynamic interference, phase differences, non-uniform wind exposure, electrical impedance mismatch, and aggregation losses can substantially alter array performance. The fourth grand challenge is therefore to establish scalable design rules linking unit-level performance to array-level energy delivery.

Near-term research should quantify wake interactions, unit spacing, orientation, phase synchronization, electrical aggregation efficiency, and failure tolerance. Modular interfaces should allow individual units to be replaced without disassembling the complete system, particularly for building-integrated and offshore applications.

Milestones should progress from demonstrating approximately predictable output scaling in small arrays to validating modular arrays under spatially non-uniform winds. At larger scales, studies should report array efficiency relative to the sum of isolated-unit outputs, unit-to-unit variability, aggregation losses, manufacturing yield, and maintenance requirements. Scalable fabrication and standardized mechanical and electrical interfaces should become design objectives alongside output enhancement.

#### 5.2.5. From Laboratory Prototypes to Standardized and Sustainable Deployment

The final grand challenge is to establish a common basis for assessing whether wind-driven TENGs are technically, environmentally, and economically deployable. The current literature uses inconsistent wind conditions, effective-area definitions, load boundaries, power metrics, and durability protocols, while life-cycle and cost information remains sparse.

Near-term standardization should define minimum reporting requirements for wind input, device geometry, cut-in criteria, electrical load, peak versus average power, power-density normalization, storage conditions, and environmental exposure. In parallel, sustainability assessment should consider material sourcing, fluoropolymer use, wear-induced material release, recyclability, disassembly, and end-of-life recovery. Economic assessment should extend beyond device material cost to packaging, electronics, installation, maintenance, and replacement.

A practical milestone would be adoption of a minimum reporting checklist enabling independent cross-study comparison. The next stage should establish scenario-specific accelerated-aging and field-validation protocols. Ultimately, engineering demonstrations should report not only electrical performance but also service-life prediction, material recovery strategy, maintenance requirements, and application-relevant economic metrics such as lifetime-delivered energy or avoided battery replacement.

Across these five challenges, data-driven modeling and edge intelligence can serve as cross-cutting enabling tools rather than independent objectives. Machine-learning methods may assist in mapping stochastic wind inputs to optimal structural parameters, predicting degradation from multi-stressor data, and scheduling loads according to harvested-energy availability. Their value should be assessed by whether they improve energy availability, lifetime prediction, or maintenance efficiency after accounting for the additional computational and energy overhead.

## 6. Conclusions

Wind-driven TENG research is progressing from proof-of-concept energy harvesting toward scenario-oriented micro-energy systems for realistic wind environments. Their primary engineering value does not lie in competing with conventional wind turbines for bulk electricity generation, but in exploiting localized, intermittent, and otherwise difficult-to-utilize airflow to support distributed low-power electronics. Urban micro-winds, offshore environments, and low-altitude complex flows therefore represent distinct application spaces with different requirements for aerodynamic response, materials, packaging, power management, and system integration.

This review establishes a scenario-oriented framework linking wind-field constraints, architecture design, materials and interfaces, usable-energy conversion, and engineering deployment. Urban applications primarily require low-speed responsiveness, turbulence tolerance, compact integration, and low noise; offshore systems place greater emphasis on moisture resistance, corrosion protection, multi-source harvesting, and long-term unattended operation; and low-altitude systems require lightweight construction, broadband adaptation, and minimal interference with host platforms. These requirements have driven the development of flutter/flag, vortex-induced, differential and contra-rotating, wind–wave synergistic, liquid–solid, and bioinspired architectures. Importantly, no single architecture is universally optimal; performance must be evaluated against the statistical wind input, environmental constraints, and load requirements of the intended scenario.

The quantitative assessment also reveals several methodological limitations in the current literature. Reported voltage, current, power, and power density cannot always be directly compared because wind-source configurations, effective-area definitions, electrical loads, and temporal definitions of power differ substantially among studies. In particular, “cut-in wind speed” may represent mechanical onset, first detectable electrical output, or merely the lowest tested wind speed. These quantities should be distinguished from the usable-power threshold at which a conditioned system can sustain a defined load. Likewise, high open-circuit voltage or generator-side peak power should not be interpreted as equivalent to usable regulated energy.

A second major gap concerns validation maturity. Many devices proposed for urban, offshore, or low-altitude applications remain validated primarily under controlled laboratory airflow. Environmental simulations and short-term field demonstrations are increasingly reported, but evidence combining long-duration natural wind exposure, coupled environmental stressors, real-load operation, degradation tracking, and maintenance records remains limited. Similarly, laboratory cycle counts cannot yet be translated reliably into field lifetime. Future reliability assessment should therefore integrate multi-stressor accelerated aging with mechanistic degradation models and long-term field observations.

Engineering progress should consequently be judged using system-level rather than isolated device-level criteria. Power management must be evaluated across the complete TENG–rectification/energy extraction–storage–regulation–load chain. Array scaling must account for aerodynamic interference, electrical aggregation losses, and maintainability. Sustainability and economics should likewise extend beyond material selection to include fabrication complexity, packaging, service life, recyclability, installation, and maintenance. For distributed sensor applications, lifetime-delivered energy, autonomous operating duty cycle, and avoided battery replacement may be more informative than peak power or cost per watt alone.

Accordingly, five interconnected challenges define the next stage of the field: reproducing and characterizing realistic wind inputs; converting low-speed response into usable regulated energy; establishing predictable lifetime under coupled environmental stressors; scaling individual devices into manufacturable and maintainable arrays; and developing standardized, sustainable, and economically assessable deployment frameworks. Progress toward these milestones would represent a more meaningful measure of engineering readiness than further increases in isolated peak output. With coordinated advances in fluid mechanics, materials, packaging, power electronics, reliability engineering, and system design, wind-driven TENGs may develop into practical distributed micro-energy components for autonomous sensing and monitoring in locations where conventional power supply is difficult or costly.

## Figures and Tables

**Table 1:** Application requirements, structural characteristics, and key challenges of wind-driven TENGs in different wind fields.

Wind-Field Type	Typical Environmental Characteristics	Main Application Requirements	Adapted TENG Configuration	Main Advantages	Key Challenges
Urban micro-wind scenarios	Low wind speed, strong turbulence, random wind direction, limited space	Low-power sensing, building monitoring, smart infrastructure power supply	Flutter, flag, differential, and Karman vortex street coupling structures	Low start-up wind speed, easy integration, and adaptability to local turbulence	Large output fluctuations, rapid wear, and limited lifespan
Offshore wind farms	High humidity, salt spray, corrosion, biofouling, coexistence of wind and waves	Marine monitoring, buoy power supply, self-powering of marine structures	Liquid–solid contact, wind–wave-coordinated, corrosion-resistant encapsulation	Resistance to environmental erosion, multi-source energy harvesting, suitability for long-term operation	Complex encapsulation, difficult maintenance, high system stability requirements
Low-altitude complex wind fields	Strong turbulence, significant wind shear, large wind-speed fluctuations, load sensitivity	Low-altitude monitoring, distributed node power supply	Biomimetic adaptive design, coaxial converter, wideband response	Lightweight, wide wind-speed adaptability, flexible deployment	Structural fatigue, unstable output, high requirements for circuit matching

**Table 2:** Quantitative performance comparison of representative wind-driven TENGs under different wind environments.

Ref.	Architecture	Scenario	Wind-Speed Range (m/s); Lowest Type	Size/Area	*V*_oc_ and *I*_sc_	*P*_max_/ *P*_avg_; Matched Load	Power Density	Storage/ Load Demonstration	Durability	Validation; Comparability Class
[10]	Flutter	General wind energy harvesting	3.4–12; (cut-in)	h = 50 µm, L = 9 cm	297 V, 3.9 µA at 10 m/s	*P*_max_ = 0.46 mW; *P*_avg_ = 0.16 mW, at 10 m/s; 100 MΩ	127.78 mW/m^2^	Charge 33 μF to 1.09 V in 38 s	NR	L1; B
[21]	Flutter	Urban micro-wind	4–15; (min)	Device 75 × 75 × 10 mm	128.4 V at 15 m/s; NR	*P*_max_ = 0.468 mW; *P*_avg_ = 0.166 mW, at 6 m/s; 300 MΩ	NR	Charge 100 μF to 1.5 V in 140 s	970 k cycles at 15 m/s	L3; B
[24]	Flag type	General wind energy harvesting	2.27–14.4; (*v*_mech_)	100 × 70 × 0.1 mm	NR; NR	NR; 50 MΩ	Average of 45 mW/m^2^ at 14.4 m/s	Charge 47 µF to 5 V in 75 s at 13.3 m/s	300 k cycles	L1; B
[25]	Flag type	General wind energy harvesting	2–8; (min)	150 × 75 × 0.08 mm	64 V, 8 µA at 7.2 m/s	*P*_max_ = 67.8 µW at 7.2 m/s; *P*_avg_ NR; 10 MΩ	6.03 mW/m^2^	Light up 100 LEDs at 8 m/s	NR	L1; B
[26]	Dual-Rotor	General wind energy harvesting	2.2–16; (*v*_mech_)	28 × 28 × 23 cm	306 V, 32 µA at 6 m/s	*P*_max_ = 5.2 mW at 9 m/s; *P*_avg_ NR; 3 MΩ	NR	Charge 1 mF to 2 V in 4 min	3-month test	L1; B
[30]	Dual-Rotor TEHG	General wind energy harvesting	2–16; (*v*_mech_)	24 × 24 × 20 cm; 0.048 m^2^	TENG 300 V, 30 µA; EMG 4.4 V, 8.7 mA	*P*_max_: TENG 5.2 mW, 10 MΩ; EMG 11 mW; 300 Ω; *P*_avg_ NR	41.05 W/m^3^ (hybrid total)	Drive sensor for 2–3 min after 10 min charging	NR	L1; A
[33]	Karman vortex street-coupled	Gas-flow sensing	0.52–3; (cut-in)	TENG 65 × 20 × 0.22 mm, cylinder 4 cm	28 V at 3 m/s; NR	*P*_max_ NR; *P*_avg_ = 3.72 µW at 2 m/s; 7.5 MΩ	26 mW/m^2^ at 2 m/s	Charge 1 μF to 41.5 V in 120 s	500 k cycles at 1.5 m/s	L2; B
[36]	Karman vortex street-coupled	Urban random gusts energy harvesting	6–10; (min)	Shell 1.96 × 10^−3^ m^3^; single-layer area 4.5 × 10^−4^ m^2^	685 V, 145 µA at 10 m/s, 1 Hz	*P*_peak_ = 8 mW; *P*_avg_ = 0.28 mW, at 10 m/s; 5 MΩ	4.08 W/m^3^ at 10 m/s	Charge 10 mF to 5.25 V in 3 h during roadside random gusts	10 h and 5.4 × 10^4^ cycles	L3; A
[38]	Karman vortex street-coupled	General wind energy harvesting	5–12; (min)	Diameter (*D*) 60 and height (*H*) 300 mm	345 V, 32.60 µA at 12 m/s	*P*_peak_ = 0.5 mW at 8 m/s; *P*_avg_ NR; 5 MΩ	0.01072 W/m^3^ at 8 m/s	Warning light after 1300 s at 6 m/s	8 h and 5.6 × 10^4^ cycles at 2 Hz	L1; B
[43]	Liquid–Solid Contact	General wind energy harvesting	1–9; (min)	Rotor *D* = 23 cm	5.4 kV at 2.8 m/s, 106 µA at 9 m/s	NR; 130 MΩ	Peak 23.9 W/m^2^, average 4.4 W/m^2^ at 8 m/s	Charge 10 mF to 6 V in 80.5 s at 3 m/s	234 k cycles	L1; B
[45]	Wind–wave synergistic	Offshore	6–16; (min)	*D* = 112 and *H* = 118 mm	245 V, 0.8 µA	*P*_peak_ = 0.11 mW; *P*_avg_ NR; 900 MΩ	216.1 mW/m^3^	Charge 470 μF to 4.5 V in 38 min	3 h in 3.5% brine	L2; B
[48]	Wind–water synergy	smart agriculture	1-5; (min)	Wind turbine *D* = 200 and *H* = 150 mm	NR; 35.21 μA at 2 m/s	*P*_peak_ = 106.86 mW; *P*_avg_ NR; NR	242.16 mW/m^2^	Charge 47μF to 3 V in 57 s at 2 m/s	8h cycles	L3; B
[52]	Rotary, soft contact	Offshore	1.4–9; (start-up)	Electrode *D* = 200 mm	2820 V, 764.75 μA at 500 rpm	*P*_avg_ = 240.00 mW at 300 rpm; 30 MΩ	NR	Charge 15 mF to 6 V in 44 s	1.8 × 10^6^ cycles	L2; B
[61]	Dual-Rotor	General wind energy harvesting	13.1–28; (*v*_mech_)	Frame 110 × 104 mm, TENG 32 × 34 mm	NR; 25.38 μA	NR; 6 MΩ	386.89 mW/m^2^	Charge 33 μF to 3 V in 27 s	11 h and 8.45 × 10^5^ cycles	L1; B
[65]	Flow-induced-vibration	General wind energy harvesting	1.2–13.8; (cut-in)	TENG 40 × 40 mm	177 V, 6.2 μA at 2.4 m/s	NR; 40 MΩ	47.43 W/m^3^ at 2.4 m/s	Charge 47 μF to 2 V in 147 s	Open environment interval 5 months	L2; B
[72]	Galloping oscillator	General/omnidirectional wind	3–5; (min)	190 mm mast length, 48.3 mm blade width	461 V, 6 mA (3 Hz, 100 mm motor bench)	*P*_peak_ 2.1 mW / *P*_avg_ 1.2 mW (3 Hz motor bench); 100 MΩ	24.1 W/m^3^	Charge 330 mF to 2.5 V in 350 s	5 h and 50,000 cycles	L3; B
[109]	Wind-cup	Low-altitude/distributed monitoring	3–7; (*v*_mech_)	TENG rotor *D* = 150 mm	300 V, 972.63 μA at 7 m/s	NR; 700 kΩ	216.84 mW·m^−2^·(m·s^−1^)^−1^	Charge 50 mAh Li-ion in 10.37 h at 3 m/s	20,736,000 cycles, 87% retention	L3; A
[113]	Double-blade TEHG	Outdoor natural-wind demonstration	2–5; (start-up)	Single TENG area 105 cm^2^	TENG 910 V, 45 μA; EMG 236 V, 24.2 mA, at 5 m/s	TENG *P*_peak_ 4 mW, 10 MΩ; EMG *P*_peak_ 0.5 W, 5 kΩ	TENG 0.38 W/m^2^	TENG charge 220 μF to 4.5 V in 4 min	NR	L3; A
[114]	Rotary TEHG	General/variable wind	4–12; (min)	Wind blade *D* = 100 and *H*= 130 mm	TENG 700 V, 22 μA; EMG 11.5 V, 57 mA, at 12 m/s	TENG *P*_peak_ 5.7 mW, 30 MΩ; EMG *P*_peak_ 180 mW, 200 Ω	NR	PM2.5 was reduced from 538 to 31 within 66 s.	1,000,000 cycles at 12 m/s	L1; B
[124]	Wind-cup; charge pump	General wind energy harvesting	3.0–9.9; (min)	Main TENG *D* = 24 cm	560 V, 1.1 mA at 300 rpm	*P*_avg_ 115 mW at 300 rpm; 500 kΩ	0.262 W m^−2^ Hz^−1^	Charge 1 mF to 2.0 V in 1.5 s	200 h and 720,000 cycles	L1; B

**Table 3:** Comparison of wind-driven TENGs and conventional wind turbines in different application scales.

Comparison Dimensions	Conventional wind Turbines	Wind-Driven TENG
Primary objective	Electrical power generation from wind at substantial power scales	Distributed micro-energy harvesting and self-powered sensing
Typical deployment	Dedicated wind-energy installations with defined rotor swept area and structural support	Embedded, distributed, or localized installation near end devices
Wind-response characteristics	Optimized for specified operating envelopes; strongly dependent on rotor scale and inflow	Some architectures respond to low-speed/intermittent/localized airflow
Start-up/cut-in characteristics	Scale- and design-dependent	Potentially low onset threshold, but definitions vary substantially among studies
Electrical characteristics	Depend on generator scale, topology, and power electronics	High source impedance; typically high-voltage, low-current, often pulsed device-level output
Power conditioning	Mature power-electronic conversion and grid/load interfaces	Rectification, impedance matching, storage, and regulation generally required
Power scale	Substantially higher and suitable for continuous electricity generation, depending on turbine scale	Generally micro-/low-power harvesting for sensors and intermittent loads
Environmental reliability	Mature engineering solutions and established design practices; turbulence/corrosion remain design considerations	Strongly affected by humidity, contamination, wear, and packaging; long-term evidence remains limited
Maintenance	Established scheduled/predictive maintenance frameworks	Potentially simple at small scale, but long-term maintenance requirements remain insufficiently established
Technology maturity	Commercially mature with established standards	Predominantly laboratory/prototype stage with limited long-term field validation
Suitable role	Wind-based electrical energy generation	Complementary localized power source for distributed low-power electronics
Major current bottleneck	Site constraints, aerodynamic loading, cost and maintenance depending on scale	Usable power, standardization, lifetime, array scaling, packaging and field validation

## Data Availability

Data are presented in the coauthors’ research results and schematic drawings, which are available on request.

## Abbreviations

The following abbreviations are used in this manuscript:

EMG	Electromagnetic generator
FEP	Fluorinated ethylene propylene
*I*_sc_	Short-circuit current
PDMS	Polydimethylsiloxane
PENG	Piezoelectric nanogenerator
PTFE	Polytetrafluoroethylene
PV	Photovoltaic
PVDF	Polyvinylidene difluoride
SHM	Structural health monitoring
TENG	Triboelectric nanogenerator
TEHG	Triboelectric–electromagnetic hybrid generator
TPHG	Triboelectric–piezoelectric hybrid generator
TPVHG	Triboelectric–photovoltaic hybrid generator
UAV	Unmanned aerial vehicles
*V*_oc_	Open-circuit voltage
